# Weak versus Deterministic Macroscopic Realism, and Einstein–Podolsky–Rosen’s Elements of Reality

**DOI:** 10.3390/e26010011

**Published:** 2023-12-21

**Authors:** Jesse Fulton, Manushan Thenabadu, Run Yan Teh, Margaret D. Reid

**Affiliations:** Centre for Quantum Science and Technology Theory, Swinburne University of Technology, Melbourne 3122, Australia; 102518796@student.swin.edu.au (J.F.); mthenabadu@outlook.com (M.T.); rteh@swin.edu.au (R.Y.T.)

**Keywords:** quantum entanglement, quantum nonlocality, macroscopic realism, Bell inequality, Leggett-Garg inequality, element of reality

## Abstract

The violation of a Leggett–Garg inequality confirms the incompatibility between quantum mechanics and the combined premises (called macro-realism) of macroscopic realism (MR) and noninvasive measurability (NIM). Arguments can be given that the incompatibility arises because MR fails for systems in a superposition of macroscopically distinct states—or else, that NIM fails. In this paper, we consider a strong negation of macro-realism, involving superpositions of coherent states, where the NIM premise is replaced by Bell’s locality premise. We follow recent work and propose the validity of a subset of Einstein–Podolsky–Rosen (EPR) and Leggett–Garg premises, referred to as *weak macroscopic realism* (wMR). In finding consistency with wMR, we identify that the Leggett–Garg inequalities are violated because of failure of *both* MR and NIM, but also that *both* are valid in a weaker (less restrictive) sense. Weak MR is distinguished from *deterministic macroscopic realism* (dMR) by recognizing that a measurement involves a reversible unitary interaction that establishes the measurement setting. Weak MR posits that a predetermined value for the outcome of a measurement can be attributed to the system *after* the interaction, when the measurement setting is experimentally specified. An extended definition of wMR considers the “element of reality” defined by EPR for system A, where one can predict with certainty the outcome of a measurement on A by performing a measurement on system B. Weak MR posits that this element of reality exists once the unitary interaction determining the measurement setting at B has occurred. We demonstrate compatibility of systems violating Leggett–Garg inequalities with wMR but point out that dMR has been shown to be falsifiable. Other tests of wMR are proposed, the predictions of wMR agreeing with quantum mechanics. Finally, we compare wMR with macro-realism models discussed elsewhere. An argument in favour of wMR is presented: wMR resolves a potential contradiction pointed out by Leggett and Garg between failure of macro-realism and assumptions intrinsic to quantum measurement theory.

## 1. Introduction

The interpretation of the quantum superposition of two macroscopically distinguishable states has been a topic of interest for decades [1,2,3,4,5,6,7,8,9,10,11]. Schrödinger considered a superposition $|\psi_{M}\rangle =\frac{1}{\sqrt{2}}(|a\rangle +\left|d\rangle \right)$ where |a〉 and |d〉 are macroscopically distinct quantum states, distinguished by some measurement $\hat{Q}$ [1]. The outcomes are associated with macroscopically distinct physical properties, analogous to a cat alive or dead. Schrödinger explained how the standard interpretation given to a quantum superposition introduces a paradox when applied to the macroscopic system. The system is interpreted as being in neither state |a〉 or |d〉 prior to measurement $\hat{Q}$ , suggesting it is somehow simultaneously in both states, which would be “ridiculous” [1].

Leggett and Garg proposed concrete tests of macroscopic realism versus quantum mechanics [12]. They introduced *macroscopic realism* (MR) as the premise that “a system with two macroscopically distinct states available to it will at all times *be* in one or other of those states”. They considered a measurable quantity $Q_{i}$ defined for the system at time $t_{i}$ ; this quantity takes the value +1 or −1 , depending on which of the two states the system is (measured to be) in. MR posits the existence of a variable, $\lambda_{i}$ , such that the value of $\lambda_{i}$ specifies which macroscopically distinct state the system is in and hence predetermines the outcome of the measurement of $Q_{i}$ . For systems described by the macroscopic superposition state $|\psi_{M}\rangle$ , the variable $\lambda_{i}$ is a “hidden” one because quantum mechanics does not give such a predetermination. In our paper, we are careful to specify MR as a *minimally restrictive* definition, where the predetermination of the outcome $Q_{i}$ does not require full knowledge of the macroscopically distinct “states” of the system, e.g., it is not assumed that the system with $\lambda_{i}=+1$ or −1 is necessarily in state |a〉 or |d〉 , nor indeed in any particular quantum state.

In order to test MR, Leggett and Garg introduced the additional assumption of macroscopic noninvasive measurability (NIM). This assumption, however, is challenging to justify [13,14,15,16,17,18,19,20]. The combined assumptions of MR and NIM are referred to as macro-realism. By considering the two-time moments $\langle Q_{i}Q_{j}\rangle$ and assuming macro-realism, Leggett and Garg derived inequalities that are predicted by quantum mechanics to be violated for certain dynamical systems involving macroscopic superposition states [12].

There have been many demonstrations of a violation of a Leggett–Garg inequality [13,14,15,16,17,18,19,20,21,22,23,24,25,26,27,28,29,30,31,32,33]. However, many of these are microscopic realizations only. Macroscopic tests exist [14,15,18,26,27], but these have been susceptible to the criticism that the local measurements used are invasive or else require auxiliary assumptions, e.g., that the “states” given by a definite value of $\lambda_{i}$ can be prepared in the laboratory [15]. A strict test of MR would not require such assumptions.

Motivated by the need to rigorously test macroscopic realism (MR), we examine in this paper a recently proposed test of macro-realism involving superpositions of coherent states, namely, entangled cat states [34,35]. Here, a measurement of the sign $\hat{Q}\equiv \hat{S}$ of a quadrature phase amplitude $\hat{X}$ distinguishes between two coherent states |α〉 and |−α〉 where α→∞ . MR implies the outcome of $\hat{S}$ to be predetermined as either positive or negative. In this proposal, the question of there being an invasive measurement is partly resolved because the outcome for the measurement of $\hat{S}$ can be inferred from a measurement made on a spatially separated system *B*, implying that the NIM premise is justified by Bell’s assumption of locality [30,36,37,38,39,40]. The Leggett–Garg inequality becomes a Bell inequality and is referred to as the Leggett–Garg–Bell inequality. The proposal may hence be interpreted in two ways: as a Leggett–Garg test, or else as a macroscopic Bell test, in which a Bell inequality involving the hidden variables $\lambda_{i}$ is predicted to be violated. Such a test predicts violation of a Bell inequality for macroscopically coarse-grained measurements, where it is not necessary to fully resolve the amplitude $\hat{X}$ . While other macroscopic Bell-nonlocality tests have been put forward [41,42,43,44,45,46,47,48,49,50,51,52,53,54,55,56,57,58], the proposal here identifies two macroscopically distinct states that allow for the simple application of MR as considered originally by Leggett and Garg. Similar tests involving coarse-grained measurements have been proposed [35,59,60,61,62].

In this paper, our motivation is to examine whether it is possible to obtain consistency with macroscopic realism (MR), despite the fact that the Leggett–Garg–Bell inequality is violated for a macroscopic proposal with a rigorous justification of noninvasive measurability. In the Leggett–Garg interpretation of the proposal, the system *A* is examined at three consecutive times $t_{i}$ ( i=1,2,3 ). At each of these times, the system has two macroscopically distinct states available to it. In between, the system evolves dynamically, according to a unitary evolution $U_{A}\left(t\right)$ . The system is entangled with a second system *B*, which also evolves locally. At time $t_{i}$ , a measurement of $Q_{i}$ on system *B* provides the outcome for $Q_{i}$ of *A*. In the Bell interpretation of the proposed experiment, the two-time moments $\langle Q_{i}Q_{j}\rangle$ become the bipartite moments of the Bell inequality. For each system *A* and *B*, the local dynamics $U_{A}\left(t\right)$ ( $U_{B}\left(t\right)$ ) corresponds to the dynamics $U_{\theta }^{A}$ ( $U_{\phi }^{B}$ ) associated with the local choice of measurement setting θ ( ϕ ), which in the traditional Bell experiment dictates which spin component $S_{\theta }^{A}$ ( $S_{\phi }^{B}$ ) will be measured.

It has been shown in previous work that the dynamics $U_{\theta }$ associated with the choice of measurement setting θ plays a key role in understanding how it is possible to find consistency with MR. Thenabadu and Reid [34] have pointed out that MR can hold despite the violation of the Bell inequalities, if defined appropriately to take into account this dynamics. “Realism” is a concept taken to imply real properties that exist independently of, or prior to, a measurement made on a system. Hence, there are two definitions of MR, depending on whether the “measurement” is defined to include the unitary dynamics $U_{\theta }$ or not. The first is *deterministic macroscopic realism* (dMR), which can be falsified by the proposed Bell cat experiments. The second, *weak macroscopic realism* (wMR), is a weaker set of assumptions that can be posited consistently with the Bell predictions. Recently, Fulton et al. [63] extended the premises of wMR to fully account for bipartite Einstein–Podolsky–Rosen (EPR) systems where “elements of reality” may be considered [64]. They defined three parts to the wMR premise: wMR(1), wMR(2), and wMR(3).

In this paper, we demonstrate the consistency of the extended wMR premises with the predicted violation of the macroscopic Leggett–Garg–Bell inequalities, as proposed in [34]. In particular, we show how the third premise wMR(3) is implicit in quantum measurement theory, where one *indirectly* infers the result of a measurement of a physical quantity of a system *A* by a *direct* measurement on a second system, the meter (for which the measurement setting is fixed). It was raised by Leggett and Garg whether a system violating macro-realism could be a good measurement device [12]. Extending arguments put forward in [34], we show how the premise wMR (3) resolves this question. The premises of wMR lead to predictions that can be tested experimentally. In this paper, we review and summarize four such tests. The tests allow for the falsification of wMR, if the experimental results are inconsistent with the predictions of wMR. This is not expected, however, since the predictions of wMR agree with those of quantum mechanics.

Fulton et al. point out that wMR can be generalized to apply to the standard microscopic Bell systems, in which case the premise of wMR is referred to as *weak local realism* (wLR) [63]. Hence, the analyses and tests we give in this paper map on to similar analyses and tests for wLR. There is consistency of wLR with the violations of Bell inequalities in standard set-ups. This has been shown by Fulton et al. [63] and by Joseph et al., who have applied the wMR/wLR premises to examine realism in Wigner’s friend paradoxes [65]. It is emphasized that the premise of weak local realism (wLR) is a *subset* of the conditions of local realism as defined in Bell’s theorem. The premises of wMR and wLR give predictions consistent with quantum mechanics and allow for the violation of Bell inequalities. Hence, these premises do *not* rule out Bell nonlocality. Our motivation in examining the wMR/wLR premises is to probe the *degree of nonlocality* that is necessarily involved in Bell violations.

The layout of this paper is as follows. In Section 2, we summarize the premises of dMR and wMR. In Section 3 to Section 6, we review and extend the arguments put forward in [34,63]. This includes the apparent inconsistency between wMR and the completeness of quantum mechanics, given along the lines of Schrödinger’s original argument [1], in Section 3. The gedanken experiment involving entangled cat states is summarized in Section 4, and the negation of deterministic macroscopic realism is explained in Section 5. In Section 6 and Section 7, we demonstrate the consistency of wMR with the predicted violation of Leggett–Garg–Bell inequalities. We also examine the premise wMR(3) involving the assumption of an “element of reality”, showing consistency with that premise. Four tests of wMR are proposed, all of which show agreement between wMR and quantum predictions. In Section 8, we explain how the wMR(3) premise can be implemented in quantum measurement theory to resolve the inconsistency raised by Leggett and Garg [12]. Finally, in Section 8, we compare wMR with models of macroscopic realism developed by Maroney and Timpson [19,20].

## 2. Weak versus Deterministic Macroscopic Realism

**Figure 1:**
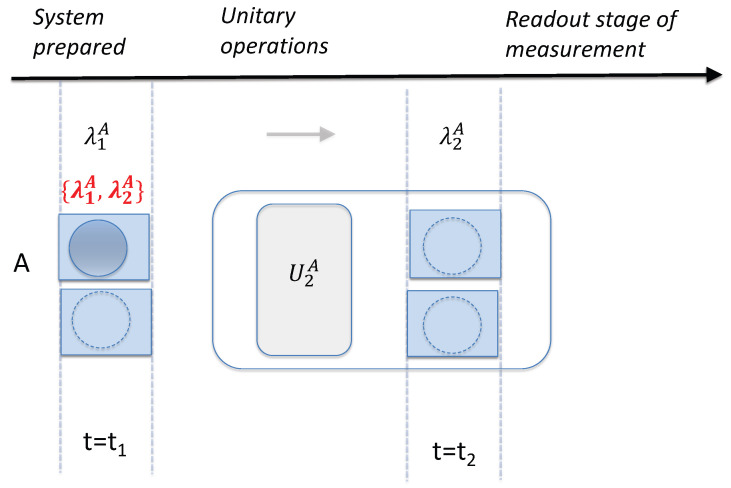
Diagram depicting the different meanings of deterministic (dMR) and weak macroscopic realism (wMR). A system is prepared at time $t_{1}$ in a state |ψ〉 . System *A* has two macroscopically distinct states available to it, depicted as a ball being in one box or the other. The diagram depicts a measurement ${\hat{S}}_{\theta }^{A}$ that can be made on the system. First, reversible interactions $U_{2}^{A}$ occur, modelled in the diagram as a shuffling of the ball between the boxes. In one view of the measurement process, these operations model the first stage of measurement that requires unitary interactions to fix the measurement setting θ . After the shuffling, at time $t_{2}$ , the location of the ball, as in which box it is in, can be determined by an observer opening the boxes. This is referred to as the “pointer”, or readout, stage of the measurement. Weak macroscopic realism (wMR) posits that the location of the ball is predetermined at times $t_{1}$ and $t_{2}$ , *after* the shuffling $U_{2}$ has occurred, just prior to the observer opening the boxes. The predetermination is represented by variables $\lambda_{i}^{A}$ that are assigned only to the system as it is defined at each time $t_{i}$ , with the value $\lambda_{i}^{A}=+1$ or −1 indicating which box the ball is located in. Alternatively, the premise dMR considers predetermined values prior to the measurement viewed in its entirety as consisting of both stages (large grey rectangle). Here, a predetermination for the outcome giving the final macroscopic state of the ball at time $t_{2}$ is assigned to the system as defined *prior* to the unitary operations, at time $t_{1}$ . This implies a simultaneous assignment of both variables $\left\{\lambda_{1}^{A},\lambda_{2}^{A}\right\}$ (in red) to the system described by |ψ〉 .

Two definitions of MR exist. The definitions depend on whether the “measurement” process includes the unitary interaction $U_{\theta }$ that determines the measurement setting θ or not (Figure 1). This stage of measurement precedes a final “pointer” measurement stage, which includes an (irreversible) readout of a meter. In the Bell experiments, the interaction $U_{\theta }$ is considered to be part of the measurement, and the relevant system is that defined *prior* to $U_{\theta }$ . In this context, *deterministic macroscopic realism* (dMR) posits that a value $\lambda_{\theta }$ predetermining the outcome of the measurement $\hat{Q}\equiv {\hat{S}}_{\theta }$ can be specified for the system as it exists prior to the measurement (which includes $U_{\theta }$ ). Here, we consider that the different sets of macroscopically distinct states $\varphi_{\lambda_{\theta }}$ (which give a definite outcome $\lambda_{\theta }$ for ${\hat{S}}_{\theta }$ ) defined for different θ can be simultaneously identified for this system. The analogy is in classical mechanics, where states with a definite *x* and *p* are defined for the system at any point of time, prior to measurement of either. It has been shown that dMR is falsifiable, according to quantum predictions that allow for a violation of macroscopic Bell inequalities [34,35].

On the other hand, the Leggett–Garg interpretation of the experiment presents a different context (Figure 1). At certain times $t_{i}$ ( i=1,2,3 ) the system is *already* prepared for the final “pointer” stage of the measurement, the unitary interaction $U_{\theta }$ being considered part of the system dynamics. In this context, the measurement basis has been fixed prior, and a less restrictive definition of MR applies. The premise of *weak macroscopic realism* (wMR) is a set of weaker assumptions that are not negated by the violation of the macroscopic Bell inequalities [34]. We consider that the system at the relevant time $t_{f}$ after the interaction $U_{\theta }$ has available to it two [or more] macroscopically distinct states, these states (which we call pointer states) corresponding to definite and distinct outcomes for the final measurement, $\hat{Q}\equiv {\hat{S}}_{\theta }$ .

The premise of wMR posits that [34]:

- *Premise wMR(1):* A predetermined value $\lambda_{\theta }$ for the outcome of the (pointer) measurement ${\hat{S}}_{\theta }$ can be ascribed to the system as it exists at the time $t_{f}$ *after* the unitary dynamics $U_{\theta }$ , at which time the measurement setting θ is fixed in the experiment. This means that the predetermination is only (necessarily) assumed for the system once it is prepared with respect to the measurement basis.
  The premise implies that the irreversible pointer stage of the measurement occurring after the unitary dynamics $U_{\theta }$ is passive, in the sense that this stage of measurement acts to reveal the value $\lambda_{\theta }$ . It is also posited that:
- *Premise wMR(2):* The value $\lambda_{\theta }$ defined for the system at the time $t_{f}$ is fixed to give the outcome of the pointer measurement (if it were to be made on the system defined at that time) and is *not changed* by any space-like separated interactions $U_{\phi }$ or events that might *then* occur at a spatially separated site.

Recently, Fulton et al. [63] have extended the definition of wMR to the situation of the Einstein, Podolsky, and Rosen (EPR) paradox [64], including Bohm’s version for spins [66]. Consider the bipartite set-up where spin measurements ${\hat{S}}_{\theta }^{A}$ and ${\hat{S}}_{\phi }^{B}$ are made on space-like separated systems *A* and *B*.

Here, wMR posits that:

- *Premise wMR(3):* Whether or not the final *pointer* measurement for the spin ${\hat{S}}_{\phi }^{B}$ has been made on one system (*B*, say) does not influence the value $\lambda_{\theta }^{A}$ for the outcome of the spin measurement of the other, *A*. Suppose one can predict with certainty the result of a measurement ( ${\hat{S}}_{\phi }$ , say) on one system (*A*, say) by making a measurement ( ${\hat{S}}_{\phi }$ , say) on the other system, *B*. The premise posits that the value for the outcome of ${\hat{S}}_{\phi }$ at *A* is specified by an “element of reality”, given by a variable $\lambda_{\phi }^{A}$ , *once* the unitary dynamics $U_{\phi }^{B}$ determining the measurement setting at *B* has taken place. The “element of reality” exists regardless of whether the unitary operation $U_{\phi }^{A}$ specifying the measurement setting at *A* has actually been carried out.

It is important to note that the value $\lambda_{\theta }$ predetermines the (macroscopic) outcome for the pointer measurement only e.g., in Schrödinger’s cat paradox [1], the cat is dead or alive in a box, prior to the observer opening the box. Similarly, in the example of Figure 1, the ball is in one or other box, prior to an observer opening the box. The predetermination $\lambda_{\theta }$ does not refer to the full “state” of the cat, or of the ball, which may specify other physical quantities. This is discussed further in Section 3 and Section 9.

Fulton et al. explain that it is possible to consider a qubit system that is not necessarily macroscopic, as in a standard Bell experiment [63]. One can consider the system at a time $t_{f}$ after which the unitary interaction $U_{\theta }$ that fixes the measurement setting has occurred. For a suitable $t_{f}$ , the system would become coupled to a macroscopic meter, in a reversible interaction, prior to any final readout. The measurement basis is specified, and at this stage the system is a macroscopic superposition of pointer states corresponding to final amplified qubit outcomes. We expect wMR to apply to the system at this time. Hence, it is possible to define *weak local realism* (wLR) as the three premises above, specifying the time $t_{f}$ .

The above premises are less restrictive than those of *local realism*, as introduced by EPR and Bell. The EPR paradox, Bell nonlocality and the Greenberger–Horne–Zeilinger (GHZ) nonlocality [67] all arise from the assumption of EPR and Bell’s strong version of local realism, where the predetermined values are assumed valid prior to the unitary interactions, $U_{\theta }^{\left(A\right)}$ and $U_{\phi }^{\left(B\right)}$ at each site [63]. Hence, the above wMR premises do *not* rule out Bell nonlocality. In fact, nonlocal effects are evident for systems satisfying wMR (and wLR) [34].

A simple picture justifying the extended wMR premises is given by considering set-ups similar to that of the three-box paradox [68,69], as in Figure 1. A ball is placed in one of three boxes at time $t_{1}$ . The state is modelled as the three-mode state |N〉|0〉|0〉 , where here |n〉 is a number state of *n* photons, the *n* quanta representing the ball. After some shuffling, the ball at time $t_{2}$ is in a superposition of being found in one of the three boxes:

(1) $$\frac{1}{\sqrt{3}}[|N\rangle |0\rangle |0\rangle +|0\rangle |N\rangle |0\rangle +|0\rangle |0\rangle |N\rangle ].$$

In this context, the unitary interaction $U_{1}$ corresponds to the shuffling, which is reversible. The pointer measurement corresponds to an observer opening the boxes, to determine the state of the ball, as in Figure 1. Further shuffling $U_{k}$ can occur, and the location of the ball examined at time $t_{k}$ after shuffling has occurred. As explained above, wMR(1) posits MR for the location of the ball, that it will be found in one of the boxes, i.e., the outcome for the ball being in a given box or not is predetermined. Hence, according to wMR, a variable $\lambda_{k}$ can be specified, the value of which indicates which box the ball will be found in, at time $t_{k}$ .

**Figure 2:**
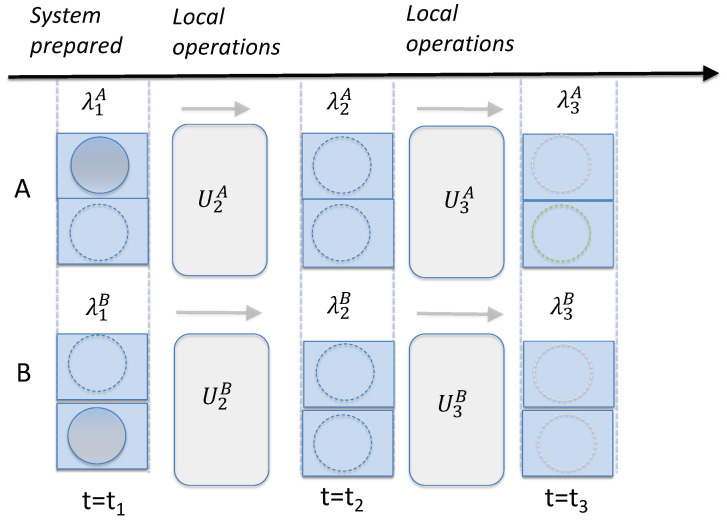
Diagram depicting the assumptions of weak macroscopic realism (wMR). Consider two separated systems *A* and *B*, each of which has two macroscopically distinct states available to it. As in Figure 1, this is depicted as a ball being in one box or the other. Reversible interactions $U_{2}^{A}$ and $U_{2}^{B}$ occur locally at each site, modelled as a shuffling of the ball between the two boxes. After the shuffling, at time $t_{2}$ , the location of each ball can be determined by opening the boxes. After time $t_{2}$ , further local shuffling operations $U_{3}^{A}$ and $U_{3}^{B}$ take place. The premise wMR(1) posits that at each time $t_{i}$ ( i=1,2,3 ), the location of each ball is predetermined prior to an observer opening the boxes. The predetermination is represented by variables $\lambda_{i}^{A}$ and $\lambda_{i}^{B}$ , assigned to each system at time $t_{i}$ . The premise wMR(2) posits that the location of the ball at one site at time $t_{i}$ is fixed once the local shuffling operation $U_{i}$ has been finalized, and is not affected by any shuffling that might then occur at the other site. The premise wMR(3) posits that if it is possible at time $t_{i}$ ( i=1,2,3 ), after the shuffling has taken place at one site (*B*, say), to predict with certainty (by determining the location of the ball at site *B*) the location of the ball of the other system (*A*) as it is placed after some specified shuffling $U_{k}^{A}$ at that site *A*, then the location of the ball at *A* after that shuffling $U_{k}^{A}$ is fixed at time $t_{i}$ . This is regardless of whether the shuffling $U_{k}^{A}$ has actually occurred at the other site *A* at time $t_{i}$ .

Figure 2 shows a set-up of two groups of two boxes, *A* and *B*, prepared in a state

(2) $${[|N\rangle |0\rangle ]}_{A}{[|N\rangle |0\rangle ]}_{B}$$

Weak MR(2) posits that once the shuffling for group *A* has finished, the location of the ball as given by $\lambda^{A}$ is fixed and cannot be changed by any shuffling that occurs at the other boxes *B*. Thirdly, wMR(3) posits that if, after some form of shuffling $U_{\theta }^{A}$ and $U_{\phi }^{B}$ at both the groups, we are able to predict with certainty which box the ball is in of group *A*, by opening the boxes at *B*, then an “element of reality” $\lambda_{\theta }^{A}$ exists for the location of the ball in group *A*. However, we can only say that this element of reality $\lambda_{\theta }^{A}$ is valid at the time $t_{\phi }$ , *once* the shuffling $U_{\phi }^{B}$ has occurred at *B*. The value $\lambda_{\theta }^{A}$ is fixed at this time $t_{\phi }$ , however, *regardless* of whether the shuffling $U_{\theta }^{A}$ has actually yet occurred at *A*. We explain in Section 8 how the premise wMR(3) justifies assumptions in quantum measurement theory, where the measurement made on a meter *B* implies the value of a physical quantity of a system *A*.

In Section 6, we give a physical example of this model of wMR by presenting an explicit interaction that realizes $U_{\theta }^{A}$ and $U_{\phi }^{B}$ . We then show how this wMR model can be consistent with the violation of Leggett–Garg and Bell inequalities. In the above example which uses number states, $U_{\theta }^{A}$ and $U_{\phi }^{B}$ can be realized at least for moderate *N* by a Hamiltonian *H* based on a Josephson coupling [35,70,71]. A more fruitful example that works in a macroscopic limit uses cat states rather than number states.

## 3. Cat States, Weak Macroscopic Realism, and Incompleteness

The objective of this paper is to analyse how weak macroscopic realism (wMR) can be consistent with violations of macroscopic Bell and Leggett–Garg inequalities. As a preliminary, before examining whether wMR can be compatible with quantum mechanics, we present a possible argument *against* wMR [34]. This concerns the inconsistency between wMR and the completeness of quantum mechanics. We review how the premise of wMR links with Schrödinger’s cat paradox [1]. This is done to highlight the importance of testing the macroscopic realism (MR), since it is not clear whether wMR is fully compatible with quantum mechanics.

Schrödinger asks whether it is feasible that a macroscopic system (a “cat”) be simultaneously in both of two macroscopically distinct states (both “dead and alive”) [1]. Weak MR counters such a claim: wMR posits that at any given time $t_{i}$ , the system is to be considered in *one or other* of the macroscopically distinct states. The essential feature of Schrödinger’s paradox is that for the system in a quantum superposition of the two macroscopically distinct states, quantum mechanics does not provide a description of what could be meant by the “states” that the “cat” is “in”. This constitutes the nature of the paradox—an apparent inconsistency between wMR and the *completeness* of quantum mechanics.

It is generally understood that those “states” for which the “cat” is either “dead” or “alive” cannot be *quantum* states. Below, we summarise the proof of this result for the system we examine in this paper: a superposition of macroscopically distinct coherent states. According to wMR, the “state” the cat is in (prior to an observer opening the box) has a predetermined value for the physical quantity *Q* that the observer measures to distinguish whether the cat is dead or alive. We show below that the superposition cannot be represented as a classical mixture of quantum states having a definite value of *Q*. The question associated with Schrödinger’s argument [1] is: If the “states” are not quantum states, what are they? Schrödinger’s argument becomes a paradox since if macroscopic realism is correct, this suggests an incompleteness of quantum mechanics: the description for the underlying states of the system is lacking.

We begin by considering the cat state [7,8,72,73,74]

(3) $$|\psi_{M}\rangle =\frac{1}{\sqrt{2}}\left(|\alpha \rangle +i|-\alpha \rangle \right)$$

of a single-mode field *A*. Here, |±α〉 are coherent states with α large and real. These states becomes macroscopically distinguishable in phase space for large α , in analogy with the “alive and dead” states, |a〉 and |d〉 , of the “cat”. Quadrature phase amplitude measurements ${\hat{X}}_{A}=\frac{1}{\sqrt{2}}\left(\hat{a}+{\hat{a}}^{†}\right)$ and ${\hat{P}}_{A}=\frac{1}{i\sqrt{2}}\left(\hat{a}-{\hat{a}}^{†}\right)$ are defined (in a rotating frame) where ${\hat{a}}^{†}$ , $\hat{a}$ are mode boson operators ( ℏ=1 ) [7]. The states |±α〉 can be distinguished by a measurement $\hat{Q}$ : here, $\hat{Q}$ is given as ${\hat{S}}^{A}$ , which has a value +1 if the outcome of ${\hat{X}}_{A}$ is positive and −1 otherwise. The outcomes +1 and −1 are analogous to the spin outcomes in a Bell experiment.

In this context, ${\hat{S}}^{A}$ is the pointer measurement. The outcome can be determined using a homodyne measurement scheme, which gives a readout on a macroscopic meter. Here, we see that the system in the state $|\psi_{M}\rangle$ has been prepared in a superposition of pointer eigenstates. The coherent states constituting the superposition are effective pointer eigenstates for large α since the outcome for ${\hat{S}}^{A}$ is given as 1 or −1 for |α〉 and |−α〉 , respectively. The state $|\psi_{M}\rangle$ is prepared for the pointer measurement ${\hat{S}}^{A}$ , with the measurement basis being eigenstates of ${\hat{S}}^{A}$ .

**Figure 3:**
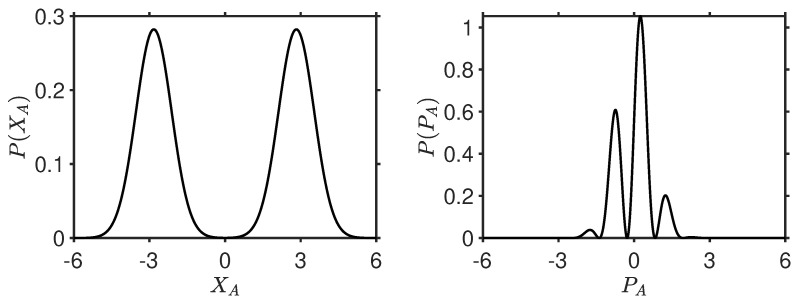
The probability distributions $P(X_{A})$ and $P(P_{A})$ for the cat state (3) with α=2 .

Weak macroscopic realism postulates that the system has a definite “spin” outcome, +1 *or* −1 , for ${\hat{S}}^{A}$ , which implies it must be in a state with a sufficiently localized outcome $X_{A}$ , for ${\hat{X}}_{A}$ . If the state is to be a *quantum* state, we arrive at a constraint on the outcomes $P_{A}$ of measurement ${\hat{P}}_{A}$ . The distribution for $X_{A}$ gives two Gaussian hills each with variance 1/2 (Figure 3). Supposing the system to be in a classical mixture of two states, one for each Gaussian, in accordance with wMR, then for each we specify the variance ${\left(\Delta X_{A}\right)}^{2}=1/2$ . If the two states are quantum states, then the uncertainty relation $\Delta X_{A}\Delta P_{A}\geq 1/2$ for each implies that the overall variance in $P_{A}$ satisfies ${\left(\Delta P_{A}\right)}^{2}\geq 1/2$ [34].

The observation of

(4) $${\left(\Delta P_{A}\right)}^{2}<1/2$$

leads to an Einstein–Podolsky–Rosen-type paradox, where, since the state consistent with wMR cannot also be consistent with the uncertainty principle, one argues either the failure of wMR or else an *incompleteness* of quantum mechanics. The underlying states posited by weak macroscopic realism (wMR), which have a definite value of the parameter ${\hat{S}}^{A}$ , cannot be quantum states. For the cat state (3), a fringe distribution is observed for ${\hat{P}}_{A}$ (Figure 3), and

(5) $${\left(\Delta P_{A}\right)}^{2}=\frac{1}{2}-2\alpha^{2}e^{-4\alpha^{2}}.$$

The paradox is obtained for all α , albeit by a vanishingly small amount for larger α [34,75,76]. The proof presented here is stronger than earlier proofs that demonstrate the incompatibility of the superposition with a classical mixture of the two coherent states, |α〉 and |−α〉 [7]. We note similar paradoxes have been given in the literature, including in [77,78].

While the original Einstein–Podolsky–Rosen (EPR) paradox revealed inconsistency between local realism and the completeness of quantum mechanics [64], Bell later proved local realism could be negated [36]. The predictions of quantum mechanics were *different* to those of local realism. This gave a resolution of the original EPR paradox, since the paradox was based on a falsifiable premise. The above argument, however, is based on weak macroscopic realism (wMR), which motivates the question of whether wMR can also be negated. We show in this paper that wMR is *not* negated by the violation of the macroscopic Bell and Leggett–Garg inequalities.

## 4. A Strong Test of Macro-Realism Using Entangled Cat States

To examine this question, we first follow [27] to demonstrate how Leggett and Garg’s macro-realism [12] can be violated for the cat state (3). In the context of the Leggett-Garg tests, the premise of MR is applied only to the system defined at the times when it is prepared for a pointer measurement (refer to Figure 2). Hence, the weaker premise of wMR suffices to define macro-realism. Macro-realism is hence defined as the combined assumptions of wMR and noninvasive measurability (NIM)—that one may determine the value of $\lambda_{i}$ as defined by wMR, without a subsequent macroscopic disturbance to the future dynamics of the system [12].

**Figure 4:**
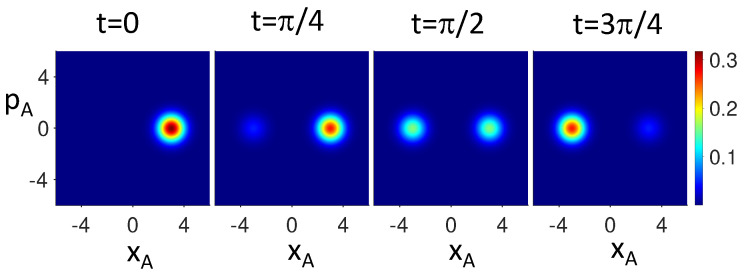
The dynamics of the system prepared in a coherent state |α〉 as it evolves for a time *t* under the action of $H_{NL}$ (Equation (6)). Here, α=3 , but the solutions apply as α→∞ . Time *t* is in units of $\Omega^{-1}$ . After an evolution time t=π/4 , the system is in the superposition (7). After evolving for a time t=π/2 , the system is in the superposition (8). The evolution is periodic and returns to the coherent state |α〉 at time t=2π . The contour plots give the *Q* function defined as $Q\left(\alpha_{0}\right)={\left|\langle \alpha_{0}|\psi \rangle \right|}^{2}/\pi =Q\left(x_{A},p_{A}\right)$ of the state |ψ〉 at time *t*. Here, $\alpha_{0}=x_{A}+ip_{A}$ .

At time $t_{1}=0$ , we consider that system *A* is prepared in |α〉 , where α is real. The system then evolves according to the nonlinear Hamiltonian

(6) $$H_{NL}=\Omega {\hat{n}}^{4}$$

where Ω is a constant and $\hat{n}={\hat{a}}^{†}\hat{a}$ . After time $t_{2}=\pi /4\Omega$ , it can be shown that the system is in the state [27,34]

(7) $$U_{\pi /8}|\alpha \rangle =e^{-i\pi /8}\left(\cos\pi /8\;|\alpha \rangle +i\sin\pi /8\;|-\alpha \rangle \right)$$

where we write $U_{\pi /8}=U_{A}\left(t_{2}\right)=e^{-iH_{NL}^{\left(A\right)}t_{2}/\hbar }$ . After further evolution, at time $t_{3}=\pi /2\Omega ,$ the system is in the cat state

(8) $$U_{\pi /4}|\alpha \rangle =\frac{e^{-i\pi /4}}{\sqrt{2}}\left(|\alpha \rangle +i|-\alpha \rangle \right)$$

where $U_{\pi /4}=U_{A}\left(t_{3}\right)$ . The dynamics is depicted in Figure 4, using the Q function [79].

At each time $t_{i}$ , we define $S_{i}$ to be the outcome of the measurement ${\hat{S}}^{A}$ . Assuming the system satisfies wMR, the value for $S_{i}$ is determined by a hidden variable $\lambda_{i}$ , with values +1 and −1 . Algebra reveals that $\langle \lambda_{1}\lambda_{2}\rangle -\langle \lambda_{1}\lambda_{3}\rangle +\langle \lambda_{2}\lambda_{3}\rangle \leq 1$ [12,21]. The two-time correlations are given as $\langle S_{i}S_{j}\rangle =\langle \lambda_{i}\lambda_{j}\rangle$ . The assumption NIM implies these could be measured since an ideal measurement of $S_{i}$ at times $t_{i}$ determines the value of $\lambda_{i}$ without subsequent disturbance to the system. Macro-realism therefore implies the Leggett–Garg inequality [12,21]

(9) $$\langle S_{1}S_{2}\rangle +\langle S_{2}S_{3}\rangle -\langle S_{1}S_{3}\rangle \leq 1.$$

Quantum mechanics predicts $\langle S_{1}S_{2}\rangle =\cos\left(\pi /4\right)$ and $\langle S_{1}S_{3}\rangle =0$ since the outcome for $S_{1}$ is known to be 1 from preparation. Establishing $\langle S_{2}S_{3}\rangle$ is not so clear because one may argue that a realistic measurement at time $t_{2}$ will affect the future dynamics. However, assuming the system is actually in one of the states |α〉 or |−α〉 at $t_{2}$ , the system at the later time $t_{3}$ will evolve to $U_{\pi /4}|\alpha \rangle$ or $U_{\pi /4}|-\alpha \rangle$ [12]. This implies $\langle S_{2}S_{3}\rangle =\cos\left(\pi /4\right)$ . The inequality (9) is violated, with the left side being $\sqrt{2}$ . One sees however from the paradox (5) that the system cannot actually quite be in either state |α〉 or |−α〉 at time $t_{2}$ , prior to measurement.

**Figure 5:**
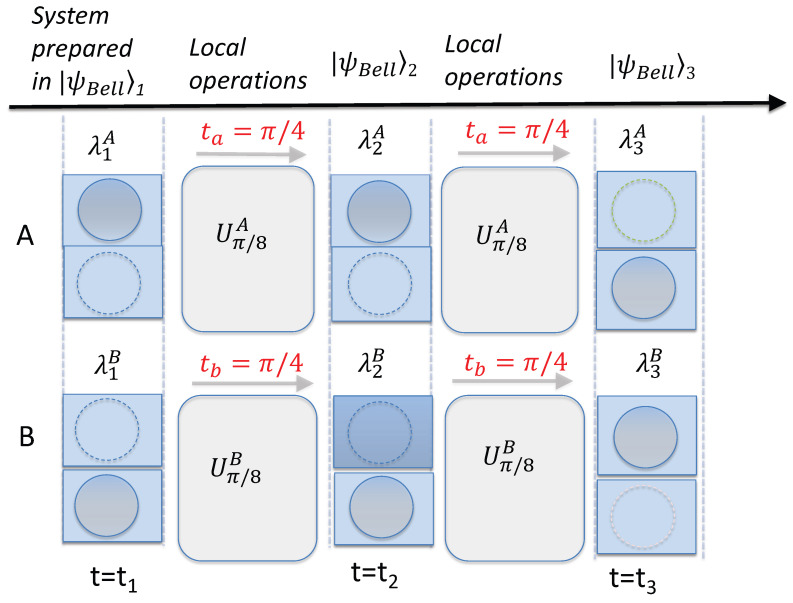
A schematic diagram of the dynamics for the measurement of spins $S_{i}^{A}$ and $S_{i}^{B}$ on the systems *A* and *B* prepared in the Bell state $|\psi_{Bell}\rangle_{1}$ (Equation (10)). The measurement of $S_{i}^{A}$ ( i=1,2,3 ) is made by measuring which state the system is in at time $t_{i}$ . This is likened to observing a ball in one or other box. The measurement $S_{2}^{A}$ is preceded by a unitary interaction $U_{2}^{A}=U_{\pi /8}^{A}$ corresponding to evolving under the action of the Hamiltonian $H_{NL}^{A}$ for time $t_{a}=\pi /4$ . Here, time is in units $\Omega^{-1}$ . Similarly, $S_{3}^{A}$ is preceded by a total evolution of $U_{3}^{A}=U_{\pi /4}^{A}$ , corresponding to evolution for time $t_{a}=\pi /2$ . The spin $S_{i}^{B}$ is measured similarly, by local interactions on system *B*. The outcomes of $S_{i}^{A}$ and $S_{i}^{B}$ are anti-correlated, with the Bell states $|\psi_{Bell}\rangle_{i}$ being created at times $t_{1}$ . Hence, the value of $S_{i}^{A}$ can be inferred indirectly from the measurement of $S_{i}^{B}$ on system *B*. In a wMR model, the location of each ball is predetermined at time $t_{i}$ , as given by $\lambda_{i}^{A}$ and $\lambda_{i}^{B}$ (Figure 2). Hence, $\lambda_{i}^{A}=-\lambda_{i}^{B}$ .

The test of macro-realism can be improved in the following manner. Consider two space-like separated systems *A* and *B* prepared at time $t_{1}=0$ in the Bell-cat state [10,34]

(10) $$|\psi_{Bell}\rangle_{1}={N\;(|\alpha \rangle }_{A}{|-\beta \rangle }_{B}{-|-\alpha \rangle }_{A}{|\beta \rangle }_{B})$$

where ${|\beta \rangle }_{B}$ is a coherent state for system *B*, $N=\frac{1}{\sqrt{2}}{\left\{1-\exp\left(-2{\left|\alpha \right|}^{2}-2{\left|\beta \right|}^{2}\right)\right\}}^{-1/2}$ , and we will take α=β with α real (Figure 5). We define the operators $\hat{X}$ , $\hat{P}$ , $\hat{S}$ , $\hat{n}$ , $H_{NL}$ , $U_{\pi /8}$ , $U_{\pi /4}$ and hidden variables $\lambda_{i}$ as above for each system *A* and *B*, denoted by a superscript *A* or *B* in each case. The systems *A* and *B* evolve independently for times $t_{a}$ and $t_{b}$ , respectively, according to local interaction Hamiltonians $H_{NL}^{A}$ and $H_{NL}^{B}$ . We define $S_{j}^{A}$ ( $S_{j}^{B}$ ) as the outcomes of the measurements ${\hat{S}}_{j}^{A}$ ( ${\hat{S_{j}}}^{B}$ ) performed after an interaction time $t_{a}=t_{j}$ ( $t_{b}=t_{j}$ ). If both systems evolve for time $t_{2}=\pi /4\Omega$ , the system is in the Bell state $|\psi_{Bell}\rangle_{2}=U_{\pi /8}^{A}U_{\pi /8}^{B}{|\psi_{Bell}\rangle }_{1}$ given by

(11) $$\begin{array}{ccc} |\psi_{Bell}\rangle_{2} & = & Ne^{-i\pi /4}(|\alpha \rangle |-\beta \rangle -|-\alpha \rangle |\beta \rangle ). \end{array}$$

If both systems evolve for time $t_{3}=\pi /2\Omega$ , the system is in the similar Bell state

(12) $$\begin{array}{ccc} |\psi_{Bell}\rangle_{3} & = & U_{\pi /4}^{A}U_{\pi /4}^{B}{|\psi_{Bell}\rangle }_{1}\\ & = & Ne^{-i\pi /2}(|\alpha \rangle |-\beta \rangle -|-\alpha \rangle |\beta \rangle ). \end{array}$$

The premise wMR assigns to *A* and *B* after interaction times $t_{a}=t_{i}$ and $t_{b}=t_{j}$ ( i,j=1,2 or 3) the hidden variables $\lambda_{i}^{A}$ and $\lambda_{j}^{B}$ (Figure 5). These take values +1 or −1 that determine (in the wMR model) the outcomes for ${\hat{S}}_{i}^{A}$ and ${\hat{S}}_{j}^{B}$ .

The failure of macro-realism is demonstrated convincingly, if one is able to perform the measurement at time $t_{2}$ without direct disturbance to the system *A*. To this end, we note the mapping that leads to the proposal of a macroscopic version of the Bell experiment [34]. As α→∞ , |α〉 and |−α〉 are orthogonal, and we map the system onto spin-qubits |↑〉 and |↓〉 , defined as eigenstates of Pauli spin ${\hat{\sigma }}_{z}^{A}$ . The rotations $U_{\pi /8}$ , $U_{\pi /4}$ , and $U_{3\pi /8}$ (defined below) become precisely the spin rotations required in the Bell experiments, realized by Stern–Gerlach analyzers or polarizing beam splitters [36,37,38].

The $S_{i}^{A}$ can be measured by taking $t_{b}=t_{i}$ and inferring the value from a measurement of $S_{i}^{B}$ at *B* (Figure 5). The anti-correlation evident in the Bell states implies $S_{i}^{A}=-S_{i}^{B}$ , and $\lambda_{i}^{A}=-\lambda_{i}^{B}$ . It is argued that this measurement is noninvasive to system *A*, based on the assumption of macroscopic Bell locality (ML). ML asserts that for space-like separated events or interactions at *A* and *B*, the events at *B* cannot change the value of the hidden variable $\lambda_{M}^{A}$ at *A*, and vice versa. This is assumed for *all* events and interactions over the time interval $t_{1}$ to $t_{3}$ , implying no macroscopic changes to the outcomes at *A* at any time $t_{i}$ due to measurement at *B* [80]. Assuming macro-realism, the inequality (9) becomes the Bell inequality [36]

(13) $$-\langle S_{1}^{B}S_{2}^{A}\rangle -\langle S_{2}^{B}S_{3}^{A}\rangle +\langle S_{1}^{B}S_{3}^{A}\rangle \leq 1.$$

**Figure 6:**
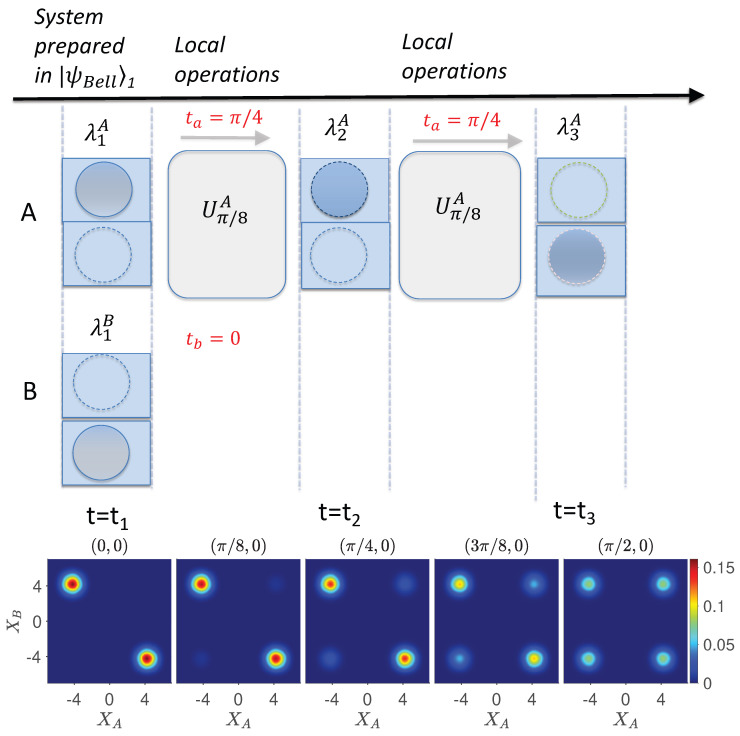
Violation of the macroscopic Leggett–Garg–Bell inequality (13) using cat states, measuring $\langle S_{1}^{B}S_{2}^{A}\rangle$ and $\langle S_{1}^{B}S_{3}^{A}\rangle$ . The diagram (top) and contour plots (lower) show the dynamics as the system prepared in the state $|\psi_{Bell}\rangle_{1}$ at time $t_{1}=0$ evolves through the measurement of $\langle S_{1}^{B}S_{2}^{A}\rangle$ and $\langle S_{1}^{B}S_{3}^{A}\rangle$ . The local systems evolve according to $H_{NL}^{A/B}$ for times $t_{a}$ and $t_{b}$ given by $\left(t_{a},t_{b}\right)$ in units of $\Omega^{-1}$ . For the measurement of $S_{1}^{B}$ , the evolution is stopped at *B* at time $t_{b}=0$ . A series of successive unitary rotations occurs at *A*. The interactions realize $U_{\pi /8}^{A}$ , and hence preparation for measurement of ${\hat{S}}_{2}^{A}$ , at time $t_{a}=\pi /4$ ; and $U_{\pi /4}^{A}$ , and hence preparation for measurement ${\hat{S}}_{3}^{A}$ , after a total interaction time of $t_{a}=\pi /2$ . Here, $t_{1}=0$ , $t_{2}=\pi /4$ , and $t_{3}=\pi /2$ . The diagram is schematic only. The contour plots show the quantum prediction for $P(X_{A},X_{B})$ at the given times. Here, α=β=3 .

**Figure 7:**
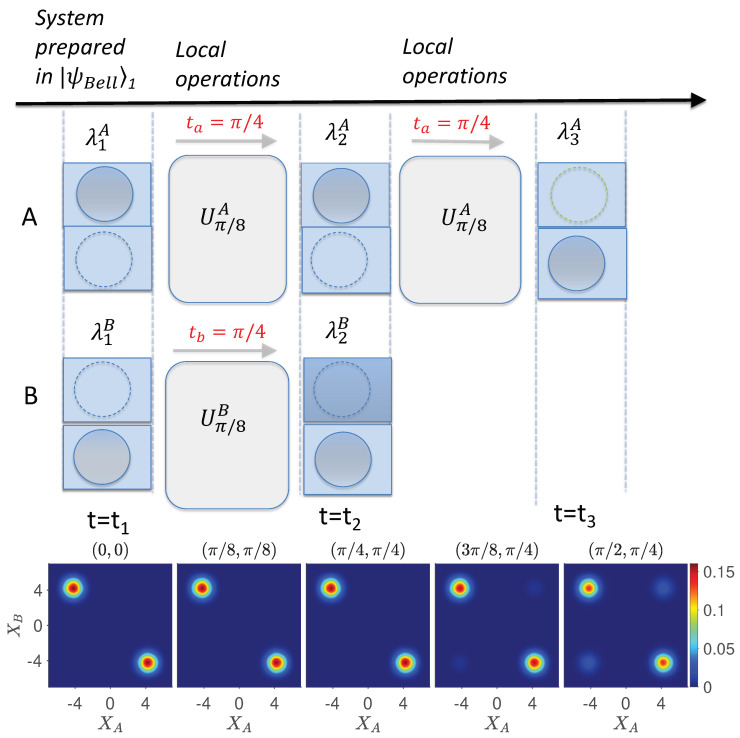
Violation of the macroscopic Leggett–Garg–Bell inequality (13) using cat states, measuring $\langle S_{2}^{B}S_{3}^{A}\rangle$ . The diagram (top) and contour plots (lower) are as for Figure 6 and show the dynamical sequence as the experimentalist measures $\langle S_{2}^{B}S_{3}^{A}\rangle$ . For the measurement of $S_{2}^{B}$ , the local evolution under $H_{NL}^{B}$ is stopped at *B* at time $t_{2}$ so that $t_{b}=\pi /4$ . For the measurement of $S_{3}^{A}$ , the local evolution under $H_{NL}^{A}$ is stopped at time $t_{a}=\pi /2$ . This moment involves a unitary rotation at each site and hence a change of measurement basis for both systems. The contour plots show the quantum prediction for $P(X_{A},X_{B})$ at the given times. The anti-correlation indicated in Figure 5 between the outcomes $S_{i}^{A}$ and $S_{i}^{B}$ is evident when $t_{a}=t_{b}$ . Here, $t_{1}=0$ , $t_{2}=\pi /4$ , and $t_{3}=\pi /2$ . Here, α=β=3 . The correlations and violations are unchanged as α , β→∞ .

The predictions based on the measurements ${\hat{X}}_{A}$ and ${\hat{X}}_{B}$ are calculated by evaluating $P(X_{A},X_{B})$ (Figure 6 and Figure 7). Where α,β>1 , the predictions for $-\langle S_{i}^{B}S_{j}^{A}\rangle$ are indistinguishable from those of $\langle S_{i}S_{j}\rangle$ given above for the predictions of the Leggett–Garg inequality (9). Violation of (13) is predicted; as for (9), the left side is $\sqrt{2}$ . The violations are valid for arbitrarily large α , β and falsify the *combined* assumptions of wMR and ML. Hence, we note that one cannot conclude the violation of wMR directly.

## 5. Falsifying Deterministic Macroscopic Realism

The violation of the Leggett–Garg–Bell inequality (9) (or (13)) does not imply falsification of weak macroscopic realism (wMR). However, one may falsify *deterministic macroscopic realism* (dMR). The inequality given by (13) is seen to be a macroscopic version of Bell’s original inequality [36,37], applied to *macroscopic* spin observables ${\hat{S}}_{j}^{A}$ and ${\hat{S}}_{j}^{B}$ . The choice between two times of evolution for each system *A* and *B* (e.g., $t_{2}$ and $t_{3}$ for *A*, and $t_{1}$ and $t_{2}$ for *B*) corresponds to a choice between two measurement settings (e.g., $\theta_{2}$ and $\theta_{3}$ for *A*, and $\phi_{1}$ and $\phi_{2}$ for *B*). This choice of unitary rotation $t_{j}$ maps in the microscopic Bell experiment to a choice of analyzer setting $\theta_{j}$ .

The Bell inequality (13) can be derived assuming *deterministic macroscopic realism*(dMR) [34]: each system *A* and *B* is *simultaneously* predetermined to be in one or other of two macroscopically distinct states, prior to the choice of measurement setting, so that *two* macroscopic hidden variables (e.g., $\lambda_{2}^{A}$ and $\lambda_{3}^{A}$ for *A*, and $\lambda_{1}^{B}$ and $\lambda_{2}^{B}$ for *B*) are ascribed to each system at the time $t_{1}$ (Figure 1). This assumption naturally incorporates ML since it is specified that $\lambda_{j}$ cannot change over the course of the unitary dynamics associated with the adjustment of measurement setting, at either site. The violation of (13) therefore falsifies dMR. The Bell inequality (13) is of the form derived by Bell in his original paper, where he considered local hidden variables of a deterministic nature [36]. In such theories, the Pauli spin component of each of the two particles is assumed to be predetermined, taking on the value of either +1 or −1 .

We may also consider the evolution of (10) for time $t_{4}=3\pi /4\Omega$ , in which case the evolved state is

(14) $$U_{3\pi /8}^{A}|\alpha \rangle =e^{i3\pi /8}(\cos3\pi /8|\alpha \rangle +i\sin3\pi /8|-\alpha \rangle ).$$

This allows for the evaluation of the familiar Clauser–Horne–Shimony–Holt Bell inequality [37,38]

(15) $$|\langle S_{1}^{B}S_{2}^{A}\rangle +\langle S_{2}^{A}S_{3}^{B}\rangle +\langle S_{3}^{B}S_{4}^{A}\rangle -\langle S_{1}^{B}S_{4}^{A}\rangle |\leq 2$$

which can also be derived from dMR. The system prepared in the Bell state (10) evolves after time $t_{4}$ to the Bell state

(16) $$\begin{array}{ccc} |\psi_{Bell}\rangle_{4} & = & U_{3\pi /8}^{A}U_{3\pi /8}^{B}{|\psi_{Bell}\rangle }_{1}\\ & = & Ne^{-i3\pi /4}(|\alpha \rangle |-\beta \rangle -|-\alpha \rangle |\beta \rangle ). \end{array}$$

This leads to predictions $\langle S_{3}^{B}S_{4}^{A}\rangle =-\cos\pi /4$ and $\langle S_{1}^{B}S_{4}^{A}\rangle =-\cos3\pi /4$ , and a violation of (15), with the left side being $2\sqrt{2}$ . Equation (15) can be viewed as the Leggett–Garg inequality

(17) $$\langle S_{1}^{A}S_{2}^{A}\rangle +\langle S_{2}^{A}S_{3}^{A}\rangle +\langle S_{3}^{A}S_{4}^{A}\rangle -\langle S_{1}^{A}S_{4}^{A}\rangle \leq 2$$

derived in [12]. Similar to (13), to obtain (15) we justify the NIM premise using ML and put $S_{i}^{B}=-S_{i}^{A}$ for times $t_{1}$ and $t_{3}$ , based on the anti-correlation of the spins for the Bell states. Alternatively, Equation (15) is seen to be a macroscopic Bell inequality, where one measures the correlation $E\left(\theta_{i},\phi_{j}\right)=\langle S_{i}^{A}S_{j}^{B}\rangle$ .

## 6. Finding Consistency of the Leggett–Garg–Bell Violations with Weak Macroscopic Realism

We now ask whether one can reconcile the violations of macro-realism and deterministic macroscopic realism (dMR) with the validity of weak macroscopic realism (wMR). To argue for consistency, it is necessary to understand that we distinguish the two stages of the measurement process and that the measurement setting is fixed experimentally by a physical, not abstract, device that requires an interaction with the system being measured.

### 6.1. Two Stages of Measurement

The first stage of measurement consists of the unitary dynamics $U_{\theta }$ that determines the experimental measurement setting. In standard Bell experiments, this corresponds to the passage of particles through a Stern–Gerlach apparatus, or else a polarizing beam splitter. In the macroscopic Bell experiments that we propose, the dynamics $U_{\theta }$ is given by the interaction of the local mode with a nonlinear medium, according to $H_{NL}$ , for a given length of time $t_{\theta }$ . The length of time determines the setting. The first stage of measurement can be reversed by applying the inverse operation $U_{\theta }^{-1}$ . After the unitary dynamics, the measurement is completed by the detection of particles or fields, and a final readout on a macroscopic meter. This constitutes the irreversible second stage of the measurement. We have referred to this second stage of the measurement as the *pointer stage* of the measurement because it leads to a reading on a meter. The different stages of measurement are modelled in Figure 1, Figure 2, Figure 3, Figure 4, Figure 5, Figure 6 and Figure 7 as a “shuffling” and as a detection, by opening a box.

The premise of wMR considers the system at time $t_{f}$ , after the first stage but prior to the second stage of measurement. Suppose the state of the system prior to the measurement is $|\psi_{M}\rangle$ . Then, at time $t_{f}$ the state has evolved and is *different*, given by

$$|\psi \left(t_{f}\right)\rangle =U_{\theta }|\psi_{M}\rangle$$

where for the macroscopic Bell test, $U_{\theta }=e^{-iH_{NL}t_{\theta }/\hbar }$ . In the standard Bell and EPR experiments, the Hamiltonian *H* is defined by the polarizing beam splitter. The premise wMR posits that the system at time $t_{f}$ can be ascribed a real property, given by $\lambda_{\theta }$ , which determines the final outcome of the measurement, if it is to be completed (without a change of measurement setting).

### 6.2. Pointer Superpositions

For wMR to apply, however, it is necessary for the state $|\psi (t_{f})\rangle$ of the system at time $t_{f}$ to have “two or more macroscopically distinct states available to it”, with these states corresponding to definite outcomes for the pointer measurement. This is true for the systems in a superposition of two macroscopically distinct coherent states, as in Equation (10). It is useful to make the analogy with the standard Bell state

(18) $$|\psi_{B}\rangle =\frac{1}{\sqrt{2}}[|↑\rangle |↓\rangle -|↓\rangle |↑\rangle ],$$

the |↑〉 and |↓〉 being eigenstates of the spin component ${\hat{S}}_{z}$ , which are the pointer states for the system prepared for the final stage of measurement of ${\hat{S}}_{z}$ . It is possible to rewrite the state in terms of the eigenstates |↑〉 and |↓〉 of ${\hat{S}}_{x}$ . However, this measurement would require a further unitary operation to be performed. The premise wMR *only* identifies a predetermined value $\lambda_{\theta }$ for the outcome of ${\hat{S}}_{\theta }$ for the system *once* it is prepared for the final pointer stage of the measurement. We refer to this as the system being prepared with respect to the measurement basis.

### 6.3. Leggett–Garg Test: Breakdown of Noninvasive Measurability

We now analyze the proposed Leggett–Garg–Bell tests of Section 4 (Figure 6 and Figure 7). The systems depicted in Figure 6 and Figure 7 at each time $t_{i}$ ( i=1,2,3 ) are prepared in a superposition of type

(19) $$\begin{array}{ccc} |\psi_{i}\rangle & = & (c_{1}{|\alpha \rangle }_{A}{|-\beta \rangle }_{B}+c_{2}{|-\alpha \rangle }_{A}{|\beta \rangle }_{B}\\ & & c_{3}{|\alpha \rangle }_{A}{|-\beta \rangle }_{B}+c_{4}{|-\alpha \rangle }_{A}{|\beta \rangle }_{B}) \end{array}$$

where the $c_{k}$ are probability amplitudes ( α , β is large). The $|\psi_{i}\rangle$ constitutes a pointer superposition. This is because the pointer measurement $\hat{S}$ can be performed directly as a homodyne measurement of $\hat{X}$ , with the phase of $\hat{X}$ being real, consistent with the phase of α as written in Equation (10). Hence, wMR can be applied to each state at time $t_{i}$ .

Hence, if wMR holds, the value of the $S_{i}^{A/B}$ is predetermined, given by $\lambda_{i}^{A/B}$ , at each time $t_{i}$ . In this case, the violations of the Leggett–Garg–Bell inequality arise because the noninvasive measurability premise (NIM), as justified by locality (ML), breaks down. Consistency with wMR is possible because the unitary dynamics

(20) $$U=e^{-iH_{NL}t/\hbar }$$

has a *finite time duration*. This means the values $\lambda_{i}^{A/B}$ can be defined at different times $t_{i}$ . This is evident in the Figure 5, Figure 6 and Figure 7, which plot the dynamics given by *U*. In Figure 5, the dynamics transforms the Bell state $|\psi_{Bell}\rangle_{1}$ prepared in the pointer basis of ${\hat{\sigma }}_{z}$ at time $t_{1}=0$ into a different Bell state $|\psi_{Bell}\rangle_{2}$ at time $t_{2}=\pi /4$ (prepared with respect to different basis), and then into a different state $|\psi_{Bell}\rangle_{3}$ at $t_{3}=\pi /2$ (prepared in the basis of ${\hat{\sigma }}_{y}$ ). The system given by the state $|\psi_{Bell}\rangle_{1}$ is not viewed to be *simultaneously* in all three pointer superpositions, in the sense that wMR cannot apply simultaneously at a single time to give a predetermination for all three measurements. One is therefore able to postulate wMR without being required to assume dMR, which fails by violation of (9), (13), and (15).

### 6.4. Bell Test: Breakdown of Local Realism

In the Bell interpretation of the Leggett–Garg–Bell experiment, the relevant measurement is that of a spin component ${\hat{S}}_{\theta }$ . Hence, the measurement process includes the entire two-stage process and the hidden variables λ are hence assigned to the system as it is defined prior to the unitary interactions $U_{\theta }$ that fix the settings θ in the experiment. Similar to those defining deterministic macroscopic realism, this means that the hidden variables pertaining to different spin components are considered simultaneously valid, prior to the measurement. This form of realism is negated by the violation of the Bell inequality. Hence, in the Bell interpretation, the violation is viewed as due to a failure of deterministic (local) realism.

At a deeper level, as we show from the Leggett–Garg interpretation, the violations are consistent with wMR (and also with wLR, refer to Section 2), hence implying the failure of noninvasive measurability (NIM). Since NIM is justified by locality, it can then be argued that the violations occur due to failure of locality. This is not inconsistent with the extended wMR premises, since the extended premises of wMR imply a *partial* locality only. This is elucidated in the next section, which examines tests of wMR.

## 7. Tests of Weak Macroscopic Realism

Careful examination of the dynamics $U_{\theta }$ associated with the measurement settings for the Leggett–Garg–Bell tests reveals further features consistent with wMR. We outline four tests of wMR, showing how the predictions for the tests are in agreement with those of quantum mechanics. We first analyse two tests that were explained in [34,81].

### 7.1. Test 1: Unitary Rotations Are Required at Both Sites to Display the Violation of the Leggett–Garg–Bell Inequality

Any theory for which wMR is valid predicts that it is the dynamics involving a unitary rotation at *both* sites that yields the violation of the inequalities (9) and (13). A similar analysis holds for the violation of (15). This is given in Ref. [63].

To show this, we examine the sequence of contour plots for $P(X_{A},X_{B})$ showing the measurement of $\langle S_{1}^{B}S_{2}^{A}\rangle$ and $\langle S_{1}^{B}S_{3}^{A}\rangle$ in Figure 6. This sequence depicts the case where there is a single rotation after $t_{1}$ . According to wMR, this sequence is consistent with macro-realism. The system is prepared in the pointer-measurement basis at time $t_{1}$ . A unitary rotation giving a change of measurement basis then takes place at *A* but not *B*. According to wMR, the system at time $t_{2}$ given by snapshot ( π/4,0 ) can be specified by *two* variables $\lambda_{2}^{A}$ and $\lambda_{1}^{B}$ that simultaneously determine the outcomes $S_{2}^{A}$ and $S_{1}^{B}$ of the measurements ${\hat{S}}^{A}$ and ${\hat{S}}^{B}$ , if performed at time $t_{2}$ . Importantly, $\lambda_{1}^{B}=-\lambda_{1}^{A}$ also determines the outcome $S_{1}^{A}$ of measurement ${\hat{S}}^{A}$ at time $t_{1}$ (given by (0,0) ). The outcome for $S_{1}^{A}$ can be determined at time $t_{2}$ without further unitary rotation because this is given by the pointer measurement at *B* at time $t_{2}$ . Hence, wMR (3) applies. Hence, by applying wMR we obtain a description for measurements made at times $t_{1}$ and $t_{2}$ that is consistent with macro-realism. Similarly, the description is consistent with Bell’s local realism, since the variables $\lambda_{2}^{A}$ and $\lambda_{1}^{B}$ predetermine the measurement outcomes for $S_{2}^{A}$ and $S_{1}^{B}$ , and the local unitary rotation at *A* does not affect the value $\lambda_{1}^{B}$ .

By contrast, the sequence of Figure 7 showing measurement of $\langle S_{2}^{B}S_{3}^{A}\rangle$ has rotations giving a change of measurement basis for *both A* and *B*. For the system given by (π/2,π/4) at time $t_{3}$ , wMR asserts the validity of variables $\lambda_{3}^{A}$ and $\lambda_{2}^{B}$ that determine the outcomes of ${\hat{S}}_{3}^{A}$ and ${\hat{S}}_{2}^{B}$ , but there is no determination of the outcome of ${\hat{S}}_{1}^{A}$ . It is the fact that the *three* variables $\lambda_{1}^{A}$ , $\lambda_{2}^{A}$ , and $\lambda_{3}^{A}$ cannot be specified simultaneously for system *A* that allows the violation of the Leggett–Garg inequality in a wMR model. The simultaneous specification is not required by wMR, because for the bipartite system it is only possible to prepare the systems in pointer bases for *two* measurements simultaneously (one at each site). The premise of wMR does not exclude that value of $\lambda_{3}^{A}$ is affected by whether or not the unitary rotation to measure $S_{2}^{B}$ occurs. Hence, wMR does not imply that the measurement of $S_{2}^{A}$ will not affect the subsequent dynamics. Hence, for this sequence of double rotations, wMR does not imply that there will be consistency with macro-realism. Similarly, we see that Bell’s local realism will not necessarily apply. Hence, there is no inconsistency between wMR and the violations of the Leggett–Garg–Bell inequality.

We now examine how this provides an experimental way to test wMR. The dynamics for the mixed state

(21) $$\rho_{mix}^{\left(AB\right)}=\frac{1}{2}(|\alpha \rangle |-\beta \rangle \langle \alpha |\langle -\beta |+|-\alpha \rangle |\beta \rangle \langle -\alpha |\langle \beta |)$$

for which a macro-realistic model holds [27] can be experimentally compared with that of $|\psi_{Bell}\rangle_{1}$ . Here, the coherent states can be distinguished by a measurement $\hat{S}$ corresponding to a dual homodyne measurement of $\hat{X}$ and $\hat{P}$ , which leaves the system in a coherent state after measurement. The mixed state also provides a local realistic model for the experiment [27]. Here, |α〉 and |β〉 are coherent states for systems *A* and *B*, and we take α=β . The system in $\rho_{mix}^{\left(AB\right)}$ is also an example of a model for which wMR holds, but it should be clear that not all wMR models imply macro-realism. According to wMR, a violation of the inequality would not arise due to the moments where there are rotations at single sites only, after the preparation at $t_{1}$ . The premise can be falsified if this is shown not to be the case. By comparing the dynamics given by the systems prepared in $\rho_{mix}^{\left(AB\right)}$ and $|\psi_{Bell}\rangle_{1}$ at time $t_{1}$ , we can show consistency with wMR for the moments where there are single rotations. A model consistent with both wMR and macro-realism exists to describe these moments, as given by $\rho_{mix}^{\left(AB\right)}$ . According to the above argument, the dynamics between systems given by $\rho_{mix}^{\left(AB\right)}$ and $|\psi_{Bell}\rangle_{1}$ will diverge where there are unitary rotations at both sites.

**Figure 8:**
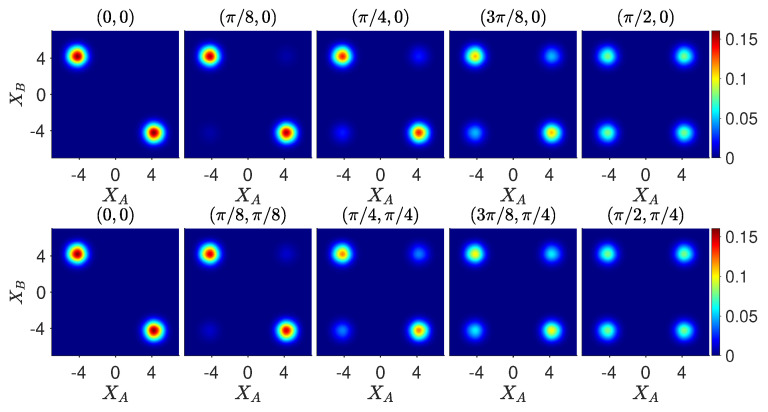
Testing weak macroscopic realism (wMR) by comparing the dynamics of $|\psi_{Bell}\rangle_{1}$ with that of a mixed state: The contours show the sequences associated with the measurements needed to test the Leggett–Garg–Bell inequality (13) as described for Figure 6 and Figure 7, except here the initial state is taken to be the nonentangled mixed state $\rho_{mix}^{\left(AB\right)}$ where no violation is possible. The top sequence is for the measurement of $\langle S_{1}^{B}S_{2}^{A}\rangle$ and $\langle S_{1}^{B}S_{3}^{A}\rangle$ , where a unitary rotation creating a change of measurement basis takes place at system *A* only. The lower sequence shows the measurement dynamics for $\langle S_{2}^{B}S_{3}^{A}\rangle$ involving unitary rotations and hence a change of measurement basis for both systems, *A* and *B*. Compared with Figure 6 and Figure 7, we see that there is no visual difference between the plots at the initial time t=0 . The top sequence involving a rotation at one site only remains visually indistinguishable from that of the entangled state shown in Figure 6. However, we see that the final state of the lower sequence involving a change of basis at each site becomes macroscopically different from the final state at time $t_{3}$ in Figure 7. This is as predicted by wMR.

For such an experiment, quantum mechanics predicts consistency with wMR. Figure 8 shows the dynamics of the measurements required to test the Leggett–Garg–Bell inequality for the system prepared initially at time $t_{1}$ in $\rho_{mix}^{\left(AB\right)}$ . The top sequence involving a rotation (change of measurement basis) at one site only is visually unaltered between the cat state $|\psi_{Bell}\rangle_{1}$ (Figure 6) and the mixture $\rho_{mix}^{\left(AB\right)}$ . The difference between the plots is of the order $e^{{-\alpha }^{2}}$ , which vanishes for the macroscopic case, where α=β→∞ [34]. By contrast, for the lower sequences where there is a rotation (change of basis) at both sites, $P(X_{A},X_{B})$ , while indistinguishable at $t_{1}=0$ , become *macroscopically different* at the later times $t_{2}$ and $t_{3}$ . This is seen when comparing the final plots of the lower sequences: the contour plot for the evolution of $\rho_{mix}^{\left(AB\right)}$ (Figure 8) is clearly different to that of $|\psi_{Bell}\rangle_{1}$ (Figure 7).

In a model where wMR is valid, it is the dynamics that occurs over the time intervals of the combination of both unitary rotations involving a change of measurement basis at each site that results in the violation of the macroscopic Leggett–Garg–Bell inequalities (13) and (15). This is consistent with calculations for violations of the Bell inequalities for microscopic spin Bell states, where it is well known that the quantum interference arising from the nonzero angles θ and ϕ is necessary to create the violation of the inequality (15). That Bell violations can arise over the course of the dynamics is consistent with predictions for Bell violations involving trajectories [80].

The dynamics given by $H_{NL}$ involves a high-order quartic nonlinearity that may not be currently readily realizable experimentally. However, the premise of weak local realism (wLR) can be tested in a similar manner using the equipment of a standard Bell experiment and comparing the Bell and mixed states $\rho_{mix}^{\left(AB\right)}$ and $|\psi_{Bell}\rangle_{1}$ , where |α〉 and |−α〉 are replaced by |↑〉 and |↓〉 as in Equation (18). The unitary interactions $U_{\theta }^{A}$ and $U_{\phi }^{B}$ are realized by a polarizing beam splitters with variable angles θ and ϕ , at each site. The system is initially prepared with respect to the measurement (pointer) basis corresponding to θ=ϕ=0 . The predictions of wLR and quantum mechanics are that where there is only one angle change (either θ or ϕ ), the moments measured for $\rho_{mix}^{\left(AB\right)}$ and $|\psi_{Bell}\rangle_{1}$ are indistinguishable. However, the moments diverge when both θ and ϕ are changed.

### 7.2. Test 2: Delaying the Pointer Stage of the Measurement

The second test of wMR concerns the timing of the second irreversible pointer (or “collapse”) stage of measurement, when the system is coupled to a detector to read out the value of $\hat{X}$ . The unitary evolution $U_{i}$ , which precedes the pointer stage of the measurement, prepares the system for the pointer measurement at time $t_{i}$ by establishing the measurement setting (i.e., measurement basis).

**Figure 9:**
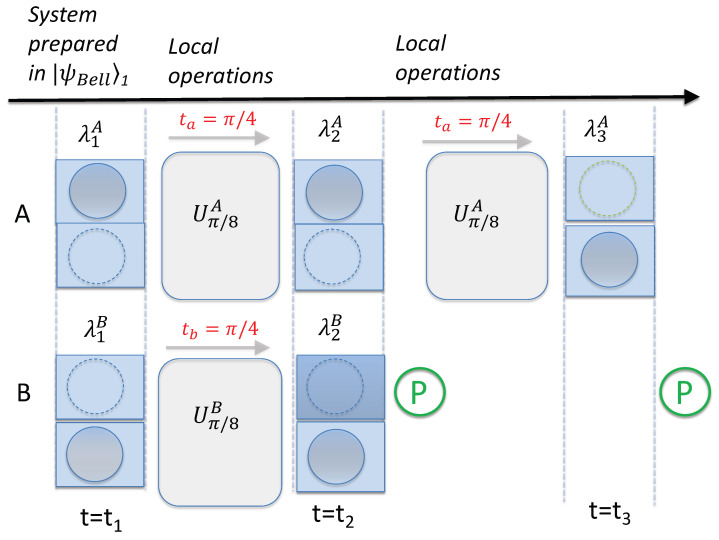
A schematic diagram of the delayed collapse test showing the consistency of weak macroscopic realism with quantum mechanics. The schematic is as for Figure 7. The two possible timings of the second irreversible readout (or pointer stage) of the measurement are depicted by the circled “P”. The readout can be made at a time before the unitary dynamics at *A*, or after, at time $t_{3}$ . In the first case, the system is viewed as having collapsed into the mixed state $\rho_{mix}^{\left(AB\right)}$ prior to $U_{\pi /8}^{A}$ being applied at time $t_{2}$ . Otherwise, the system remains in the Bell state $|\psi_{Bell}\rangle_{2}$ . We show that this makes no difference to the moments $\langle S_{2}^{B}S_{3}^{A}\rangle$ as calculated in a wMR model, which is consistent with the quantum prediction. Here, time is in units of $\Omega^{-1}$ .

In a model where wMR is valid, the hidden variable $\lambda_{i}^{A}$ for system *A* ( i=1,2,3 ) is fixed in value ( +1 or −1 ) at time $t_{i}$ , after application of the unitary evolution $U_{i}^{A}$ that prepares the basis at *A*. This gives a record of $\lambda_{i}^{A}$ at the particular time $t_{i}$ − that cannot be changed by the future collapse of *B*. There is also no retrocausality: the value defined at that time is fixed as a record. The same analysis applies for the variable $\lambda_{j}^{B}$ . If we consider the measurement of $S_{3}^{A}$ and $S_{2}^{B}$ as in Figure 9, then $\langle S_{2}^{B}S_{3}^{A}\rangle =\langle \lambda_{2}^{B}\lambda_{3}^{A}\rangle$ . Let us consider the premises of wMR defined in Section 2. According to wMR(2), the value of $\lambda_{2}^{B}$ is not affected by the subsequent unitary interactions at *A*. There is no change in the value of $\lambda_{2}^{B}$ depending on when the readout stage of measurement at *B* occurs, i.e., whether it occurs before or after the evolution $U_{3}^{A}$ and readout at *A*. Similarly, according to wMR(3), the value of $\lambda_{3}^{A}$ is not influenced by the final pointer measurement at *B*. Hence, the measured moment $\langle S_{2}^{B}S_{3}^{A}\rangle$ is unchanged by the timing of the pointer measurement at *B*.

Quantum mechanics predicts consistency with wMR: It is possible to delay the collapse stage of the measurement ${\hat{S}}_{j}^{B}$ at *B* by any amount of time after the measurement at *A*, i.e., after $t_{3}$ , and it makes no detectable difference to $P(X_{A},X_{B})$ [34]. The details of the calculation are given in [34]. To summarize, we consider the set-up of Figure 9, where at time $t_{2}$ , the unitary evolution $U_{2}^{B}$ at *B* has taken place. We first consider that the collapse at *B* takes place immediately (prior to the evolution $U_{3}^{A}$ and measurement $S_{3}^{A}$ at *A*) so that the system is prepared in the mixed state $\rho_{mix}^{\left(AB\right)}$ (Equation (21)). System *A* then evolves according to a unitary evolution $U_{3}^{A}$ . $P(X_{A},X_{B})$ is calculated for this final state. This compares with the evaluation of $P(X_{A},X_{B})$ assuming the collapse at *B* does not take place until after the evolution $U_{3}^{A}$ . Then, the system at time $t_{2}$ is in the Bell state $|\psi_{Bell}\rangle_{2}$ , and $P(X_{A},X_{B})$ is evaluated after the application of $U_{3}^{A}$ on this state. The difference between the predictions is of order $e^{-{\left|\alpha \right|}^{2}}$ , vanishing in the limit of large α . This is as expected since from the time $t_{2}$ there is only one further unitary rotation, $U_{3}^{A}$ , at the single site *A*. The macroscopic nonclassical effects that distinguish $\rho_{mix}^{\left(A,B\right)}$ from $|\psi_{Bell}\rangle_{2}$ require unitary rotations at both sites.

The experiment to test wMR would involve measuring and comparing the moments $\langle S_{2}^{A}S_{3}^{A}\rangle$ with the change in timing of the final readout at *B*. As for the Test 1, the proposed Test 2 can be performed experimentally by adapting a standard Bell set-up, to test weak local realism.

### 7.3. Test 3: Delayed-Choice Experiments: Non-Retrocausality and Extra Dimensions

The premise of wMR specifies a fixed value $\lambda_{i}$ for the outcome of the measurement ${\hat{S}}_{i}$ at the given time $t_{i}$ . This cannot be changed by any future event. One might therefore ask whether delayed-choice experiments would falsify wMR?

The following argument in favor to reject wMR could be made. We consider the set-up of Figure 6 and Figure 7, to measure $\langle S_{1}^{A}S_{3}^{A}\rangle$ or $\langle S_{2}^{A}S_{3}^{A}\rangle$ . The measurement $S_{3}^{A}$ is by a direct measurement on system *A* after a suitable interaction time $t_{a}$ , whereas $S_{1}^{A}$ or $S_{2}^{A}$ is measured indirectly, by the measurement on the correlated system *B*. The choice of $t_{b}$ hence determines whether $S_{1}^{A}$ or $S_{2}^{A}$ will be inferred. The joint probabilities $P(X_{A},X_{B})$ depend on the *local* interaction times $t_{a}$ and $t_{b}$ only. Hence, one can delay the choice $t_{b}$ to measure $S_{1}^{A}$ or $S_{2}^{A}$ until *after* the final detection at system *A*, at time $t_{3}$ . This might suggest that the measurement at *B* is necessarily noninvasive of the dynamics at *A*, and hence that the ensuing violation of the Leggett–Garg inequality (9) is due to failure of wMR.

However, as with delayed-choice experiments for spin-qubits [82,83,84,85,86,87], this interpretation can be countered [88]: A careful analysis reveals that in order to measure $\langle S_{2}^{A}S_{3}^{A}\rangle$ , a unitary evolution *U* occurs at both sites after time $t_{2}$ : There is the evolution $U_{3}^{A}$ at *A*, and then $U_{2}^{B}$ at *B*. It is shown in [34,81] that the violation of the Leggett–Garg inequality can hence be explained as a failure of dMR and be found consistent with wMR. The delayed-choice tests do not lead to the falsification of wMR.

**Figure 10:**
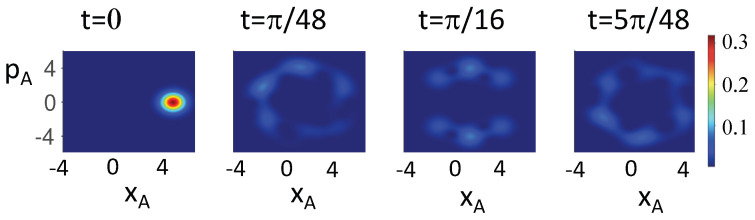
The dynamics of the system prepared in a coherent state |α〉 as it evolves for a time *t* under the action of $H_{NL}$ (Equation (6)), as in Figure 4. Here, α=4 . Time *t* is in units of $\Omega^{-1}$ . It is evident that for the times intermediate between the $t_{i}$ ( i=1,2,3 ) where $t_{1}=\pi /4$ , $t_{2}=\pi /2$ , and $t_{3}=3\pi /4$ as shown in Figure 5, Figure 6 and Figure 7, the system is not necessarily in a simple superposition of the two states, |α〉 and |−α〉 . Here, the extra dimensions allowed by the continuous phase space representation are important in portraying the intermediate dynamics. The intermediate times correspond to the “shuffling” process, where there is a change of the measurement basis. The contour plots give the *Q* function defined as $Q\left(\alpha_{0}\right)={\left|\langle \alpha_{0}|\psi \rangle \right|}^{2}/\pi =Q\left(x_{A},p_{A}\right)$ of the state |ψ〉 at time *t*. Here, $\alpha_{0}=x_{A}+ip_{A}$ .

On the other hand, a modified delayed-choice experiment can lead to a falsification of a subset of weak MR models. The delayed-choice Wheeler–Chaves–Lemos–Pienaar experiment [88,89,90] falsifies all *two-dimensional non-retrocausal models* for a two-state system, described by qubits {|↑〉,|↓〉} . Using the mapping (7) and considering the unitary rotations $U_{\theta }^{A}$ where θ is a multiple of π/8 , it is possible to map the microscopic spin experiment involving {|↑〉,|↓〉} onto one involving the macroscopic qubits {|α〉,|−α〉} [81]. Assuming the predictions of quantum mechanics are verified, this enables a falsification of all *two-dimensional non-retrocausal models* based on the macroscopic qubits {|α〉,|−α〉} [81]. This contradicts the premise of wMR, which gives a non-retrocausal model—but *only* if we are restricted to two-dimensional models. The falsification of wMR is avoided by noting the *extra dimensions* associated with the continuous-variable phase-space representation of the cat-states, which are measurable. The system as it evolves under the action of $H_{NL}$ from the superposition $|\psi_{M}\rangle$ of Equation (3) is not restricted to the two-state basis {|α〉,|−α〉} . The full phase-space is necessary to describe the evolution, as is evident by Figure 10.

The original realizations of the Wheeler–Chaves–Lemos–Pienaar gedanken experiment involved microscopic photonic qubits {|↑〉,|↓〉} , with the unitary rotations being realized by beam splitters [88,89,90]. As such, these experiments rule out two-dimensional models of weak local realism (wLR) since wLR is a non-retrocausal model.

### 7.4. Test 4: EPR’s Elements of Reality Are Justified after the Setting Dynamics

We now consider the postulate wMR(3) in the set-up of the EPR experiment. This extends the earlier work of [34]. Here, we test for the consistency between the quantum predictions and the weaker modified concept of EPR’s “elements of reality”, as specified by the premise wMR(3).

First, we review the original definition of EPR’s “elements of reality”. Examining the state (10), we see that the Bohm-EPR paradox for spin applies. At the given time $t_{i}$ , the outcome of the measurement ${\hat{S}}_{i}^{A}$ at *A* can be predicted with certainty by the measurement of ${\hat{S}}_{i}^{B}$ on system *B*. We see that $S_{i}^{A}=-S_{i}^{B}$ . EPR’s original premises posit that an *element of reality* $\lambda_{i}^{A}$ exists for system *A* at this time [64]. In EPR’s original formalism, this value predetermines the outcome of the measurement $S_{i}^{A}$ if measured directly at *A*, *regardless of whether the measurement at B is performed or not*, because the outcome at *A* can be predicted in principle by establishing the measurement at *B* and nothing at *B* can influence the system at *A*, according to EPR’s assumption of locality. The value for the element of reality is $\lambda_{i}^{A}=-\lambda_{i}^{B}$ and can be determined by finalizing the measurement at *B*.

As is well known, these original EPR “elements of reality” can be falsified. The assumption of the $\lambda_{i}^{A}$ can be applied to (non-commuting) measurements at the different times, $t_{i}$ and $t_{j}$ , as explained in Section 5. Hence, the EPR premises lead to the premise of dMR, which is falsified by the Bell test violating inequalities (13) or (15) [36].

However, the weaker premise wMR(3) is *not* falsified. This is because it refers to system *B* at time $t_{i}$ *after* any appropriate unitary interaction $U^{B}$ has taken place at *B*, to finalize the measurement setting at *B*, i.e., to prepare the system for the final pointer measurement ${\hat{S}}^{B}$ . This suggests that the “elements of reality” may apply, in certain circumstances, as specified by the premise wMR(3). As explained by Clauser and Shimony [38], the importance of the dynamics associated with the choice of measurement setting was commented on by Bohr, in his reply to Einstein, Podolsky, and Rosen [91]. Clauser and Shimony interpret Bohr’s argument to imply that “*it is incorrect to say that system 2 is not disturbed by the experimentalist’s option to measure a rather than $a^{\prime }$ on system 1.*” Here, *a* and $a^{\prime }$ refer to the measurement settings.

**Figure 11:**
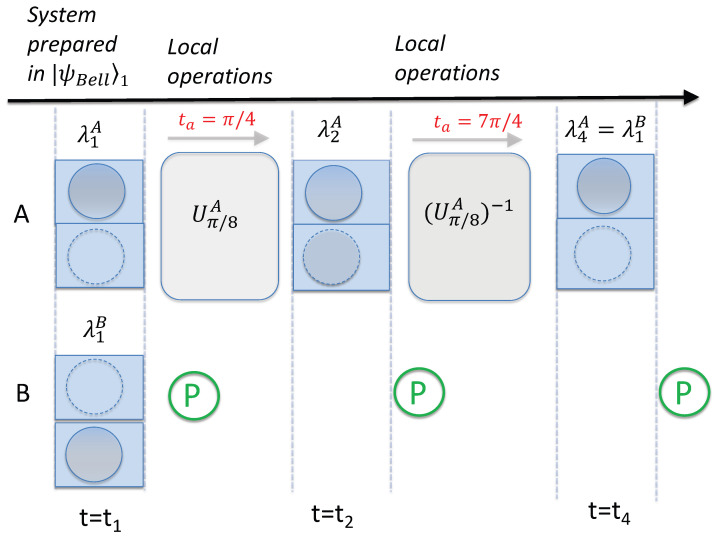
A schematic diagram showing the meaning of the premise wMR(3). At time $t_{1}$ , the outcome of the measurement of $S_{1}^{A}$ can be predicted with certainty by a measurement $S_{1}^{B}$ on system *B*. The measurement setting is fixed at *B* at time $t_{1}$ , meaning that any local “shuffling” have been carried out. According to wMR(1), the outcome of the pointer measurement *P* for $S_{1}^{B}$ is hence determined at time $t_{1}$ and can be represented by $\lambda_{1}^{B}$ . According to wMR(3), the prediction for $S_{1}^{A}$ is predetermined (and can be represented as an “element of reality”) for system *A*, at time $t_{1}$ . The predetermined value is designated by the variable $\lambda_{1}^{A}=\lambda_{1}^{B}$ . However, according to wMR(3), the prediction $\lambda_{1}^{B}$ holds to predetermine the outcome of $S_{1}^{A}$ , as long as the measurement setting at *B* remains fixed (no further shuffling at *B*), even if there are further unitary interactions at *A* as in the diagram, where the local system *A* evolves for time t=π/4 according to $U_{\pi /8}^{A}$ . The value of $\lambda_{1}^{B}$ gives the prediction for $S_{1}^{A}$ at time $t_{2}$ , even though a unitary interaction ${\left(U_{\pi /8}^{A}\right)}^{-1}$ is required at *A* to carry out the measurement of $S_{1}^{A}$ . In this case, a reversal of $U_{\pi /8}^{A}$ is achieved by evolving system *A* for a further time $t_{a}=7\pi /4$ . The measurement of $S_{1}^{A}$ at time $t_{4}$ would be completed by a local pointer measurement, which would yield the value $\lambda_{1}^{B}=\lambda_{1}^{A}$ . The predetermination for $S_{1}^{A}$ exists, irrespective of when or whether the pointer measurement *P* at *B* is made. Here, time is in units of $\Omega^{-1}$ .

Figure 7 depicts the dynamics showing the consistency of the quantum predictions with the premise wMR(3). At time $t_{1}=t_{0}=0$ , system *B* has been prepared for the final detection and readout of spin ${\hat{S}}_{1}^{B}$ . The wMR postulate is that system *B* has the predetermined value $\lambda_{1}^{B}$ for the outcome of that measurement. According to wMR(3), this also gives the value for the measurement ${\hat{S}}_{1}^{A}$ at *A*, regardless of any further unitary interactions $U_{j}^{A}$ that might take place at *A*. It would be possible to rotate the measurement basis at *A* to prepare for the measurement ${\hat{S}}_{2}^{A}$ by evolving system *A* according to $U_{\pi /8}^{A}$ but keeping *B* unchanged. This would create a new state. However, this would not change the “element of reality” $\lambda_{1}^{A}=-\lambda_{1}^{B}$ for the outcome of measurement ${\hat{S}}_{1}^{A}$ . The measurement ${\hat{S}}_{1}^{A}$ can still be made by reversing the unitary operation $U_{\pi /8}^{A}$ and performing the final part of the measurement, ${\hat{X}}_{A}$ . In other words, after a further time, the system evolves according to the dynamics ${\left(U_{\pi /8}^{A}\right)}^{-1}$ , and the prediction according to quantum mechanics is that the results of the measurements at *A* and *B* remain anticorrelated. According to wMR(3), the value $\lambda_{1}^{B}$ gives the prediction for ${\hat{S}}_{1}^{A}$ at *A*, regardless of any local reversible unitary interactions, such as $U_{\pi /8}^{A}$ at *A* (Figure 11).

An experimental test of the premise wMR(3) can be performed. This is depicted in Figure 11. The value for the element of reality $\lambda_{1}^{B}$ can be obtained by a final measurement readout at the separated system *B*. This gives the prediction for ${\hat{S}}_{1}^{A}$ . The value for ${\hat{S}}_{1}^{A}$ can be confirmed to be correct, both without and then with the unitary rotation $U_{\pi /8}^{A}$ followed by its reversal. The evolution of $H_{NL}$ is periodic, and the reversal is hence achieved with time $t_{a}=2\pi -\pi /4$ (in units where Ω=1 ). Similarly, the prediction can be verified for different times of the pointer measurement at *B*. Quantum mechanics gives predictions consistent with those of wMR.

It might be considered obvious that the value measured for ${\hat{S}}_{1}^{B}$ of system *B* fixes the outcome for ${\hat{S}}_{1}^{A}$ . This is the basis for quantum measurement, when a system *A* observable is measured by coupling to a meter. The question that the premise of wMR(3) addresses is as follows: *At what time is* the outcome for ${\hat{S}}_{1}^{A}$ actually fixed? In summary, the premise wMR(3) posits that it is fixed at the time after which the measurement setting at *B* is fixed.

## 8. Weak Macroscopic Realism, Weak Local Realism, and Quantum Measurement

The concept of weak macroscopic realism (wMR) may resolve questions about the nature of the quantum measurement. This can be put forward as an argument in favor of wMR. A fundamental question is how to understand the connection between “realism” and states such as

(22) $$|\psi_{M}\rangle =\frac{1}{\sqrt{2}}{(|↑\rangle }_{A}{|\beta \rangle }_{B}-{|↓\rangle }_{A}{|-\beta \rangle }_{B})$$

formed at time $t_{k}$ after a macroscopic measurement device *B* interacts with a microsystem *A* prepared in the superposition

(23) $$|\psi_{A}\rangle =\frac{1}{\sqrt{2}}{(|↑\rangle }_{A}-{|↓\rangle }_{A})$$

of the two eigenstates of ${\hat{\sigma }}_{z}^{A}$ . Here, |β〉 and |−β〉 are coherent states of the system *B* (we take β to be real). The readout of ${\hat{S}}^{B}$ gives the measured value of ${\hat{\sigma }}_{z}^{A}$ . A model for an interaction $H_{M}$ that evolves (23) into (22) has been presented [92,93,94]. In that model, the meter system *B* is prepared initially in a coherent state |γ〉 . The phase of β is determined by the phase γ of the initial coherent state.

A fundamental question arises: At what point in the measurement process does the value for the outcome of the measurement emerge? How is realism connected to measurement [95,96,97]?

### 8.1. Weak Local Realism

As summarised in Section 2, it becomes apparent that the wMR premises can be applied to spin systems described by {|↑〉,|↓〉} , even where the spin states may not be macroscopically distinct. This is because there is a direct mapping between the systems violating the Leggett–Garg-Bell inequalities for {|α〉,|−α〉} and those that violate for {|↑〉,|↓〉} . In this case, we refer to the premises of weak macroscopic realism as *weak local realism* (wLR). An explanation has been given in [63]. These premises are weaker (less restrictive) than those of local realism defined by Bell and are not negated by violations of Bell inequalities. Section 7.3 suggests a wMR model in which the spin states be completed by extra dimensions.

### 8.2. Schrödinger’s Cat Paradox

How to understand the entangled state (22) was the paradox put forward by Schrödinger in his essay [1]. It is often supposed that the value of the outcome of ${\hat{\sigma }}_{z}^{A}$ is not determined prior to the measurement of it, but in the wMR and wLR models, this is overstated. In these models, the value for the outcome of the spin ${\hat{\sigma }}_{z}^{A}$ of *A* is specified at, or by, the time $t_{k}$ of the creation of the entangled system-meter system in the state (22). This is because the *measurement basis (setting) for the meter has been specified by the interaction $H_{M}$ *, through the phase of the coherent field. After $t_{k}$ , only the “pointer” measurement corresponding to the detection of the amplitude ${\hat{X}}_{B}$ of the meter is required to complete the measurement. According to wMR, there is a predetermined value $\lambda_{M}^{B}$ for the amplitude ${\hat{X}}_{B}$ of the macroscopic meter *B* at this time. According to wMR(3), this value is an “element of reality” for the outcome of ${\hat{\sigma }}_{z}^{A}$ at *A* since it gives the value if it were to be measured directly. Hence, in the wMR and wLR models, the value for the outcome of the measurement can be assigned to system *A* at time $t_{k}$ , prior to the final detection and readout, since the measurement setting for *A* has been established.

A local unitary interaction at *A* can be further applied to change the measurement setting for the spin measurement at *A*. However, we have seen that in the wMR (wLR) models this makes no difference to the outcome $\lambda_{M}^{B}$ specified for spin ${\hat{\sigma }}_{z}^{A}$ at *A*, as given by the “element of reality” defined at *B*. The result for spin ${\hat{\sigma }}_{z}^{A}$ is specified by the meter and would be verified if the measurement ${\hat{\sigma }}_{z}^{A}$ at *A* is actually performed. If a local unitary interaction has since changed the measurement setting at *A*, then for the spin ${\hat{\sigma }}_{z}^{A}$ to *actually* be measured, a further unitary interaction giving a reversal takes place.

Similarly, if a local unitary interaction at *B* is implemented while keeping system *A* unchanged, it does not change the element of reality for system *B* that is implied by the fact the outcome ${\hat{X}}_{B}$ can be inferred by the spin measurement at *A*. On the other hand, if unitary interactions are implemented to change the measurement settings at both *A* and *B*, then in the wMR (wLR) models, we can no longer suppose that the value of $\lambda_{M}^{B}$ applies to a future measurement.

### 8.3. Leggett and Garg’s Question about the State of the Measurement Device That Violates Macro-Realism

In their paper [12], Leggett and Garg consider states such as (22). They explain that the violations of macro-realism should “*not be formally in conflict with the arguments so often given in discussions of the quantum theory of measurement to the effect that once a microsystem has interacted with a realistic measuring device, the device (and, if necessary, the microsystem) behave as if it were in a definite (and noninvasively measurable) macroscopic state*”.

They also suggest that system $|\psi_{M}\rangle$ , if violating macrorealism, would not be a suitable measuring device, by continuing: “*The macroscopic systems suitable for a macroscopic quantum coherence experiment are certainly not able to be measuring devices, at least under the conditions specified. But such a result might cause us to think a great deal harder about the significance of “as if”!*”

We extend the analysis of the statements of Leggett and Garg for this system, given in [34]. We examine the first statement of Leggett and Garg. The premise of wMR does indeed imply a “definite macroscopic state” for the measuring device, given by system *B* in (22), in the sense that there is a predetermination of the outcome of ${\hat{S}}^{B}$ . This is because the measurement setting ${\hat{S}}^{B}$ has been specified for the system in the state (22), by the interaction $H_{M}$ . This interaction specifies the phase of the coherent-state amplitude β . All that is required to complete the measurement is a pointer measurement, involving a detection of the amplitude ${\hat{X}}_{B}$ .

Assuming wLR, the microsystem *A* also has a definite value for the outcome of ${\hat{\sigma }}_{z}^{A}$ —but only when prepared (after the choice of measurement setting) in a superposition with respect to the pointer bases of ${\hat{\sigma }}_{z}^{A}$ and ${\hat{S}}^{B}$ . When we write the original state $|\psi_{A}\rangle$ of (23), it is not specified whether or not the measurement basis has been determined experimentally. However, we see as explained above that once entangled with the meter as in the state (22), there is a definite value for the outcome of ${\hat{\sigma }}_{z}^{A}$ . This is because the measurement setting for the microsystem is specified.

**Figure 12:**
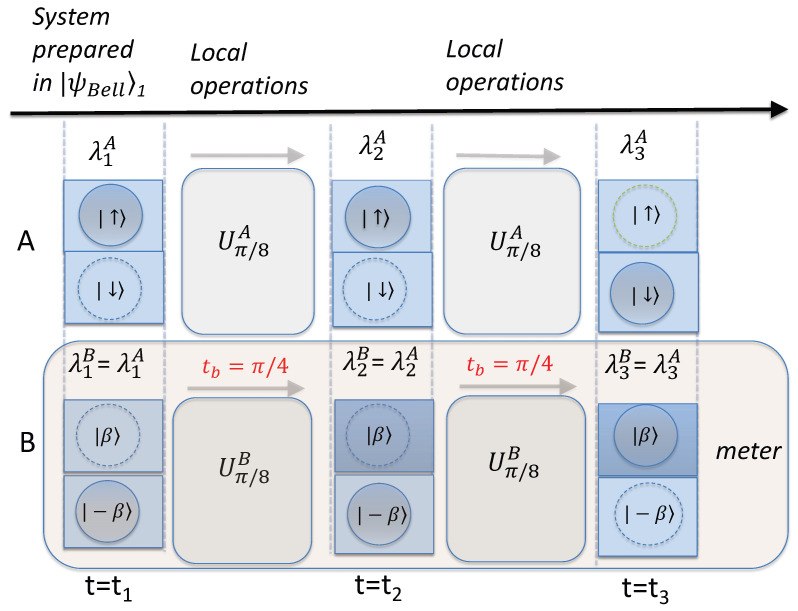
A schematic diagram depicting how a measurement device (a meter) that violates macro-realism can be consistent with the concept of being in a “definite and non-invasively macroscopic state”. The systems are prepared at time $t_{1}$ in the entangled Bell-type state $|\psi_{M}\rangle$ (22). System *B* acts as a meter for the observable $S_{1}^{A}$ of system *A* because the value of $S_{1}^{A}$ can be inferred with certainty by measuring $S_{1}^{B}$ . If we evolve the systems according to local unitary operations $U_{\pi /8}^{A}$ and $U_{\pi /8}^{B}$ (refer text), then the value of $S_{2}^{A}$ can be inferred by the outcome of $S_{2}^{A}$ . Similarly, systems *A* and *B* can be evolved according to the further operations $U_{\pi /8}^{A}$ and $U_{\pi /8}^{B}$ to allow for the measurement of $S_{3}^{A}$ . As in Figure 6 and Figure 7, these measurements allow for the violation of macro-realism (for either system *A* or *B*). Yet, according to wMR(1), system *B* at each time $t_{i}$ ( i=1,2,3 ) is in a definite macroscopic state with a predetermined value $\lambda_{M}^{B}=\lambda_{i}^{B}$ for the outcome of $S_{i}^{B}$ , which gives the outcome for the measurement $S_{i}^{A}$ if performed. According to wMR, the value $\lambda_{M}^{B}=\lambda_{i}^{B}$ of the meter system *B* is not changed once the setting (shuffling) is fixed at *B* and is hence “non-invasively measurable” (e.g., by measuring system *A*). However, the *full* state description of system *B* is not definite and non-invasively measurable.

Hence, there is no conflict with Leggett and Garg’s statement “that the device behaves *as if* it were in a definite macroscopic state”. The basis for the spin at *A* is determined to be fixed (as the eigenstates of ${\hat{\sigma }}_{z}^{A}$ ) because the coherent states that act as the meter (when ${\hat{X}}_{B}$ is measured) have a definite fixed phase, and no further rotation $U^{B}$ is necessary (Figure 12). In the wMR model, the value $\lambda_{M}^{A}$ for ${\hat{\sigma }}_{z}^{A}$ is determined by that of $\lambda_{M}^{B}$ : $\lambda_{M}^{A}=\lambda_{M}^{B}$ . There is an element of reality $\lambda_{M}^{A}$ for the result of the spin ${\hat{\sigma }}_{z}^{A}$ of *A*, for the system in the entangled meter-system state. We note that in the wMR model, the value $\lambda_{M}^{A}=\lambda_{M}^{B}$ is the value of the spin ${\hat{\sigma }}_{z}^{A}$ if measured directly and that this value $\lambda_{M}^{B}$ can be revealed by a direct readout at *B*. Importantly, in the wMR model, the predicted value for the measurement of ${\hat{\sigma }}_{z}^{\left(A\right)}$ is *always* $\lambda_{M}^{A}=\lambda_{M}^{B}$ , even if there are further local unitary interactions $U^{A}$ (and their reversals) at *A*.

Leggett and Garg also state that the meter should be “noninvasively measurable”. We note that in accordance with the premise wMR(2), the value $\lambda_{M}^{B}$ that determines whether the meter *B* will be found with positive or negative amplitude is fixed, *provided* there is no further change of measurement basis for the meter (i.e., no further “shuffling” at *B*). At any point, the value of $\lambda_{M}^{B}$ can be determined by a measurement on system *A* (with an appropriate choice of measurement basis at *A*). According to wMR(2), the value of $\lambda_{M}^{B}$ would not be changed. In this sense, the wMR model satisfies Leggett and Garg’s condition (refer Figure 12).

Now, we turn to examine Leggett and Garg’s second statement. Noting that the same predictions are given on mapping {|−α〉,|α〉} onto {|↑〉,|↓〉} for (10) in Section 4, we see that macro-realism is indeed violated for the macroscopic measuring device *B* (provided the unitary rotations are carried out appropriately for the system *A*). Yet, contrary to what may be suggested by Leggett and Garg’s statements, for theories where wMR (or wLR) is valid, we argue that there is *no conflict* with the arguments of quantum measurement theory. This is because system *B* has a definite value $\lambda_{M}^{B}$ for the outcome of ${\hat{S}}^{B}$ . System *A* of $|\psi_{M}\rangle$ also has a definite value $\lambda_{M}^{A}$ for the outcome of ${\hat{\sigma }}_{z}$ when prepared in (22).

In short, contrary to what might be supposed, the argument that the systems (22) can be considered to have definite real values $\lambda_{M}^{B}$ and $\lambda_{M}^{A}$ does not contradict the Bell violations (for example, of (15)) since we have shown consistency with wMR (and also with wLR, in the microscopic case) for such violations. The values, however, refer *only* to systems prepared appropriately at a given time in a superposition with respect to pointer-bases of ${\hat{\sigma }}_{z}^{A}$ and ${\hat{S}}^{B}$ . Suppose one could specify that *A* (prior to the measurement interaction) was prepared appropriately in $|\psi_{A}\rangle$ of (23), for the pointer-basis of ${\hat{\sigma }}_{z}^{A}$ : According to wLR, system *A* has a definite value $\lambda^{A}$ for the outcome of ${\hat{\sigma }}_{z}^{A}$ . Provided $\lambda^{A}=\lambda_{M}$ , it can then be argued that system $|\psi_{M}\rangle$ is a suitable measuring device.

Leggett and Garg have suggested in their statement that there may be a “conflict” if we are to consider that measurement devices allow for violations of macro-realism. We have shown that there is not since violations of macro-realism can be consistent with (weak) macroscopic realism. There is, however, a “conflict”, if we consider the meaning of a “*definite macroscopic state*”. The conflict arises when we consider the consequence of the wMR and wLR assumptions, summarized in Section 3, concerning the completeness of quantum mechanics: It is well known that the systems cannot be considered to be in either quantum state ${|↑\rangle }_{A}$ or ${|↓\rangle }_{A}$ , or in |β〉 or |−β〉 , since superpositions of these states are distinguishable from classical mixtures of them [7]. If wMR is valid, it is unclear which *“state”* each of the systems are actually *in*. An analysis of ontological states defining macroscopic realism has been given by Maroney and Timpson [19,20], which motivates the Section 9.

## 9. Comparison with Other Models of Macroscopic Realism

Our conclusions are consistent with those of Maroney [19] and Maroney and Timpson [20], who in analyzing tests of macro-realism have argued that violations of the Leggett–Garg inequalities arise from a nonclassical form of measurement disturbance and do not necessarily imply failure of macroscopic realism. Maroney and Timpson considered three models of macroscopic realism, which they refer to as macro-realism models. First, they defined the *operational eigenstates of a property* as “those preparations [of the system] which determinately fix the value of the property”. In our context, these are preparations of the system for which there is a predetermined value for the outcome of the measurement ${\hat{S}}_{\theta }$ .

The three models of macroscopic realism considered are operational eigenstate mixture macro-realism (OEM-MR), operational eigenstate support macro-realism (OES-MR), and supra eigenstate support macro-realism (SES-MR). Maroney and Timpson argued that only OEM-MR gives the strict form of macro-realism that necessarily leads to the derivation of the Leggett–Garg inequality. We next examine each of these models for consistency with weak macroscopic realism as defined in this paper.

### 9.1. Operational Eigenstate Mixture Macro-Realism

The OEM-MR specifies that the system after preparation (after the unitary interactions $U_{\theta }$ ) is in a mixture of the operational eigenstates. This model is negated by the violation of Leggett–Garg inequalities and is compatible with wMR but is a stronger model than required by wMR.

An example of an OEM-MR model is the mixed state (21), where system *A* prior to the measurement $\hat{S}$ can be considered to be either in the state |α〉 or |−α〉 . Here, $\hat{S}$ is selected as the dual measurement of amplitudes $\hat{X}$ and $\hat{P}$ . The coherent states become eigenstates of $\hat{S}$ (for large α ) and are also operational eigenstates. Here, $\hat{S}$ distinguishes between the two coherent states (for large α ). The measurement can be shown to not change the system placed in one or other of the coherent states.

Maroney analyzes the three-box paradox, where MR would imply that a ball placed in a superposition of being in one of three boxes is always actually in one of the boxes [19]. A Condition (III) is satisfied that a measurement made on the system where a ball is placed in a box is confirmed to be non-disturbing to the state of the system. This confirms that the measurement is non-invasive for operational eigenstates. Maroney claims that “*An intuition lurking alongside the idea that the ball is always in one, and only in one, of the boxes, is that whenever the ball is in a given box, it behaves exactly as it appears to behave when it is observed to be in that box. This runs into difficulties, for when the ball’s location is observed, it is in an operational eigenstate. This rather natural idea of macro-realism would lead to operational eigenstate mixture macro-realism*…”.

Weak macroscopic realism (wMR) does not imply OEM-MR since it is not assumed that the state of the system before and after the measurement are the same. This is evident from the analysis of Section 2, where it is proved for the cat state (3) that, if wMR holds, the system prior to the measurement $\hat{S}$ cannot be in one or other quantum state that is an eigenstate of $\hat{S}$ . The states of the cat system satisfying wMR are necessarily different before and after the measurement.

### 9.2. Operational Eigenstate Support Macro-Realism

The OES-MR and SES-MR models consider the system to be, prior to measurement $\hat{S}$ , in a mixture of ontic states that have definite predetermined values for the measurement $\hat{S}$ . For the three-box paradox, the measurement $\hat{S}$ corresponds to observing whether the ball is found in a given box. Operational eigenstate support macrorealism (OES-MR) constrains the ontic states to be in the support of the operational eigenstates.

For OES-MR, Maroney comments about the application to the three-box paradox [19]: “*The unobserved ball’s ontic state is always one that can occur when the ball is being observed. However, the price is that those ontic states must now be behaving differently to their appearances. Neither positive- nor negative-result non-invasiveness will be possible, even for operational eigenstates. While the observed behaviour of the ball, determinately placed in one box while Bob checks Condition (III), is showing no detectable disturbance, something must nevertheless be undergoing change, below the level of appearances, as a result of Bob’s measurements. This change takes place even when Bob is only interacting with a different box: placing the ball in Box 1, then opening the empty Box 2, somehow disturbs the ball in Box 1 in an unobservable way. But when the system is prepared as in a quantum superposition, and the ball is not being directly observed, these same disturbances emerge and lead to observable consequences*”.

Positive-result and negative-result non-invasiveness refers to the measurement having no disturbance to the system when the system is directly measured as a ball being observed in a box and indirectly measured, as in a ball not being observed in a box. The OES-MR model allows for nonlocality since there can be a disturbance to the ’state’ of the ball in Box 1, when an empty Box 2 is observed.

Our work expands the analysis of Maroney for the OES-MR model. Here, wMR posits that the observation of a ball not being in Box 2 would not change the variable $\lambda_{M}^{\left(1\right)}$ that predetermines the outcome of the measurement on the Box 1. However, the state of the system can change. If there is a further unitary interaction at Box 1, and at Box 2, so that measurement settings change, observable paradoxes can occur.

We note that wMR counters OES-MR since it is not true that “the unobserved ball’s ontic state is always one that can occur when the ball is being observed”. The “observed” state of the system is identifiable as a quantum state, and for the cat state (3), we have seen that the assumption of wMR implies the system cannot be in a quantum state prior to measurement.

### 9.3. Supra Eigenstate Support Macro-Realism

The supra eigenstate support macro-realism (SES-MR) model also considers the system to be, prior to measurement $\hat{S}$ , in a mixture of ontic states that have definite predetermined values for measurement $\hat{S}$ . Different to the OES-MR model, however, the SES-MR model allows for novel ontic states that cannot be prepared quantum mechanically.

Maroney, in examining the third SES-MR model, states that: “*Supra eigenstate support macro-realism takes the opposite route. Operational eigenstates do not appear to be disturbed by Bob’s measurements, and it may be maintained that the ontic states in their support are not, in fact disturbed. However, when the ball is prepared through a quantum superposition, it may now be in an ontic state that does not appear in any operational eigenstate. When it is not being observed, the ball can behave differently*”.

The premise of wMR gives support to the SES-MR model of macroscopic realism proposed by Maroney and Timpson. These authors also present the de Broglie–Bohm model [66] as an example of an SES-MR model [19]. In a recent paper [98], the wMR premises have been shown to be consistent with a model for realism based on the *Q* function [99,100,101]. An analysis of that model suggests ontic states that cannot be compatible with “prepared” or “observed” states [102].

## 10. Discussion and Conclusions

In this paper, we have examined a macroscopic version of a Leggett–Garg and Bell test presented earlier [34], in which the spin states |↑〉 and |↓〉 are realized by coherent states |α〉 and |−α〉 , with α→∞ , and the unitary interactions determining the measurement settings θ in the Bell test, normally realized by polarizing beam splitters or Stern–Gerlach apparatuses, are realized by local nonlinear interactions $U_{\theta }=e^{-iH_{NL}t/\hbar }$ . In particular, the set-up allows the noninvasive measurability premise of the Leggett–Garg inequalities to be replaced by that of Bell’s locality assumption. The corresponding Bell test is macroscopic, meaning that the Bell premises combine the assumptions of macroscopic realism (MR) and locality at a macroscopic level (ML).

Earlier work showed how MR, if defined *deterministically*, can be falsified [34,61]. Macroscopic realism applies to a system with “two or more macroscopically distinct states available to it” and posits the system to be in one of those states [12], thereby implying that a measurement ${\hat{S}}_{\theta }$ distinguishing between the states has a predetermined outcome. *Deterministic macroscopic realism* posits a predetermination of the outcome *prior* to the entire measurement dynamics, including the implementation of $U_{\theta }$ , and is a stronger (more restrictive) assumption. In such a model, as in classical mechanics, it is assumed that there are a set of macroscopically distinct states giving a definite outcome for ${\hat{S}}_{\theta }$ , which can be identified for the system prior to the time at which $U_{\theta }$ is implemented.

Violations of Bell inequalities are explained generally as a failure of “local realism”, or of “local hidden variables” [36]. The violations exclude that there can be hidden variables satisfying the Einstein–Podolsky–Rosen (EPR) premises. EPR’s “elements of reality” are negated by Bell violations. The macroscopic version of the Bell test motivates a deeper consideration of the meaning of local realism and, in particular, of the EPR premises since any rejection of macroscopic realism would be a more startling conclusion than the rejection of local realism at the microscopic level.

Our conclusion is that MR is not contradicted by the Bell violations and can be viewed consistently with the violations, *if* defined in a less restrictive way, as *weak macroscopic realism* (wMR). Weak macroscopic realism has been proposed earlier, and recent work gives an extension of the definition to the bipartite set-up of EPR [63]. The earlier work showed the consistency of the macroscopic Bell violations with a subset of the wMR premises [34]. Here, we show the consistency of the macroscopic Bell violations with the expanded definition of wMR. The authors of [34] proposed three tests of wMR, where the results would be consistent with wMR according to quantum mechanics. We present a fourth test, involving EPR’s “elements of reality”.

The consequence of our work is a model consistent with quantum mechanics, in which there is an understanding of when the EPR “elements of reality” may be valid. The “elements of reality” apply in the context of the system defined after the unitary interaction $U_{\theta }$ has been carried out in the experiment. In this model, we see that the violation of the Bell inequalities occurs due to a *combination* of a failure of realism and locality. On the other hand, both a weaker version of realism and a weaker version of locality apply: The system has a real property for the outcome of the measurement ${\hat{S}}_{\theta }$ after the implementation of $U_{\theta }$ . Also, the system *A* has an “element of reality” for the outcome of ${\hat{S}}_{\theta }$ , *if* the outcome of ${\hat{S}}_{\theta }$ at *A* can be predicted with certainty by measurement ${\hat{S}}_{\phi }$ on a second system *B*—but this applies only *once* the implementation of the unitary interaction $U_{\phi }$ at *B* has taken place.

A justification for wMR is given on considering the nature of quantum measurement. Consider system *A* for which ${\hat{S}}_{\theta }$ is being measured, by a coupling to a macroscopic meter, which we refer to as system *B*. This is a situation for which EPR’s “element of reality” applies because one can predict with certainty the outcome of the measurement on system *A* by performing a measurement on meter *B*. While EPR’s traditional “elements of reality” can be negated, this particular “element of reality” is justified by wMR because the coupling interaction is such that the measurement basis θ has already been specified. Hence, wMR provides a way to resolve possible inconsistencies relating to macroscopic realism and quantum measurement, as highlighted by Leggett and Garg [12].

While the motivation for examining wMR is to arrive at a model allowing some form of macroscopic reality, the mapping between the microscopic and macroscopic Leggett-Garg-Bell tests ensures that a similar definition, *weak local realism* (wLR) [63], can be applied to the original set-up of Bell involving the microscopic spin states |↑〉 and |↓〉 . The original Bell violations can be explained consistently with wLR. A justification for wLR can also be given based on the argument that in the microscopic tests, at time after the unitary dynamics $U_{\theta }$ establishing the measurement setting θ , there will be some form of amplification, such as a coupling to a meter. Hence, wMR can be applied at this time [63].

It is interesting to consider the possibility of an experiment. While the predictions of wMR are consistent with those of quantum mechanics, four tests of wMR have been presented, which motivates an experiment. Two-mode entangled cat states have been experimentally realized [10,103]. However, it may be challenging to realize $U_{\theta }$ . The Bell example with cat states was presented because of the strength of the conclusions that follow from a Bell violation and because of the simplicity of the argument from a theoretical viewpoint. Other macroscopic realizations of quantum correlations can be considered, however [104]. This includes the continuous variable correlations of the Einstein–Podolsky–Rosen (EPR) paradox, which are measured by homodyne detection, with the measurement setting θ being a phase shift [105,106,107]. Here, wMR can be examined, since amplification can be modelled as taking place after the choice of phase shift, but prior to a final detection, as part of the measurement process [98]. Also, set-ups are possible where amplification takes place prior to the implementation of the phase shift θ [108,109,110], so that macroscopic states can be defined and both dMR and wMR posited for the system. We also note that mesoscopic quantum correlations have been achieved for distinct atomic systems [111,112,113,114,115,116,117,118]. In particular, EPR correlations involving atomic clouds have been measured, including where the measurement setting is adjustable locally [118]. This has led to a realization of Schrödinger’s description of the EPR paradox, in which there is a simultaneous measurement of two non-commuting observables, *x* and *p* [119]. An analysis of wMR testing for consistency with such EPR correlations would be interesting.

In conclusion, we have outlined how a weak form of local realism can be consistent with realism at a macroscopic level, despite violations of macroscopic Bell inequalities. Yet, Schrödinger’s argument is that there is inconsistency between (weak) macroscopic realism and the completeness of quantum mechanics [1]: if (weak) macroscopic realism is valid, then for a system in a macroscopic superposition, which state is the system in prior to detection (since the system cannot be viewed as being in any quantum state)? This motivates an analysis of deeper models and interpretations of quantum mechanics [96,98,99,100,101,102,120,121,122,123,124,125,126,127,128,129,130,131,132,133,134,135,136,137]. Bohm’s theory is an example of a nonlocal realistic model for quantum mechanics [120,121]. Other models exist, based on retrocausal mechanisms [99,126,130,131,134]. 

## Data Availability

Data are contained within the article.
